# Roles of the leader-trailer helix and antitermination complex in biogenesis of the 30S ribosomal subunit

**DOI:** 10.1093/nar/gkad316

**Published:** 2023-04-27

**Authors:** Benjamin R Warner, Ralf Bundschuh, Kurt Fredrick

**Affiliations:** Department of Microbiology, The Ohio State University, Columbus, OH 43210, USA; Center for RNA Biology, The Ohio State University, Columbus, OH 43210, USA; Center for RNA Biology, The Ohio State University, Columbus, OH 43210, USA; Department of Physics, The Ohio State University, Columbus, OH 43210, USA; Department of Chemistry and Biochemistry, The Ohio State University, Columbus, OH 43210, USA; Division of Hematology, Department of Internal Medicine, The Ohio State University, Columbus,OH 43210, USA; Department of Microbiology, The Ohio State University, Columbus, OH 43210, USA; Center for RNA Biology, The Ohio State University, Columbus, OH 43210, USA

## Abstract

Ribosome biogenesis occurs co-transcriptionally and entails rRNA folding, ribosomal protein binding, rRNA processing, and rRNA modification. In most bacteria, the 16S, 23S and 5S rRNAs are co-transcribed, often with one or more tRNAs. Transcription involves a modified RNA polymerase, called the antitermination complex, which forms in response to *cis*-acting elements (*boxB, boxA* and *boxC*) in the nascent pre-rRNA. Sequences flanking the rRNAs are complementary and form long helices known as leader-trailer helices. Here, we employed an orthogonal translation system to interrogate the functional roles of these RNA elements in 30S subunit biogenesis in *Escherichia coli*. Mutations that disrupt the leader-trailer helix caused complete loss of translation activity, indicating that this helix is absolutely essential for active subunit formation in the cell. Mutations of *boxA* also reduced translation activity, but by only 2- to 3-fold, suggesting a smaller role for the antitermination complex. Similarly modest drops in activity were seen upon deletion of either or both of two leader helices, termed here hA and hB. Interestingly, subunits formed in the absence of these leader features exhibited defects in translational fidelity. These data suggest that the antitermination complex and precursor RNA elements help to ensure quality control during ribosome biogenesis.

## Graphical Abstract

**Graphical Abstract:**
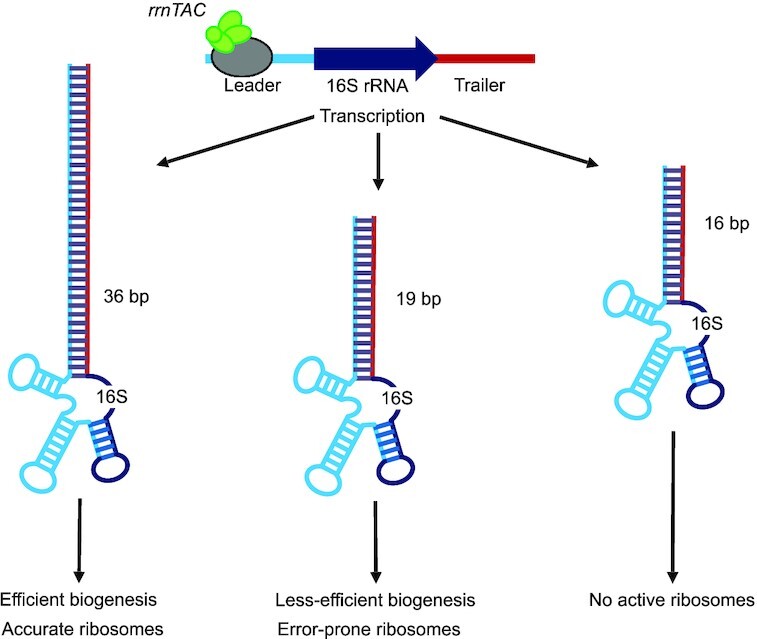
A critical role for the leader-trailer helix in 30S subunit biogenesis.

## INTRODUCTION

The ribosome is a two-subunit enzyme responsible for protein synthesis. In *Escherichia coli*, the large subunit consists of the 23S rRNA [2904 nucleotides (nt)], 5S rRNA (120 nt) and 34 ribosomal (r) proteins, while the small subunit consists of the 16S rRNA (1542 nt) and 21 r proteins. Ribosome assembly occurs co-transcriptionally and entails folding of the rRNA, binding of r proteins, modification of nucleotides and amino acid side chains, and processing of the rRNA by various RNases. Active ribosomal subunits can be reconstituted *in vitro* by mixing their constituent components (1,2), suggesting that these molecules themselves store all the necessary information for assembly. While subunits can be reconstituted *in vitro*, the process occurs slowly and requires non-physiological conditions. In the cell, non-ribosomal proteins known as assembly factors (AFs) facilitate the process (3–6). There are multiple types of AFs, including ribonucleoprotein-binding proteins, helicases, chaperones, RNases, modification enzymes and GTPases. AFs are believed to facilitate ribosome assembly by preventing kinetic traps that would otherwise inhibit the process.

In most bacteria, the 16S, 23S and 5S rRNAs are co-transcribed along with one or more tRNAs (7). In *Escherichia coli*, there are seven of these operons, varying mainly with respect to the tRNA gene content (8). Flanking each rRNA are complementary sequences that form extended helices, known as leader-trailer helices (9). RNase III recognizes and cleaves the leader-trailer helices, releasing secondary precursors (pre-16S rRNA, pre-23S rRNA and pre-5S rRNA) from the primary transcript (10,11). Other RNases subsequently work to trim both ends of the precursor rRNAs, resulting in the mature length rRNAs (12). Processing and assembly are intimately intertwined, as reflected by the fact that precursor rRNAs are present in late-stage assembly intermediates (13). Moreover, immature particles containing precursor rRNAs accumulate when individual AFs are absent or when particular processing events are blocked (14,15).

Transcription of certain long operons entails an elaborated form of RNA polymerase termed the transcription antitermination complex (TAC), which exhibits enhanced elongation processivity. Antitermination has been most extensively characterized in lambdoid bacteriophages, which use phage-encoded protein N (or Q) to regulate TAC formation. N-utilization (*nut*) sites, composed of *cis*-acting RNA elements *boxA*, *boxB* and *boxC* of the nascent RNA chain, recruit N and host-encoded Nus (N-utilization substance) factors to RNA polymerase (RNAP) (16,17). Protein N binds *boxB*, a conserved stem-loop (18–21), and NusB binds *boxA* (22). Factors NusA, NusG, and S10 (also called NusE) then associate, forming the N-TAC (16,18,23–26). N-TAC is highly resistant to Rho-dependent and intrinsic terminators (27–30) and elongates the RNA chain at twice the rate of unmodified RNAP (31).

A similar mechanism of antitermination is operational during transcription of the ribosomal RNA (*rrn*) operons. In this case, modification of RNAP involves NusA, NusB, NusE(S10), NusG, SuhB, and S4, and the resulting antitermination complex is termed *rrn*TAC (32–36). One set of *cis-*acting elements—*boxB*, *boxA*, and *boxC* (5′ to 3′)—lies at the beginning of the operon, just after promoter P2, and another lies upstream of the 23S rRNA gene (22,37). Of the RNA motifs, *boxA* is most important, being both necessary and sufficient for antitermination factor recruitment and *rrn*TAC formation (38). The presence of *boxB* in the context of *rrn* is puzzling, since there are no host-encoded versions of N, and the role of *boxC* remains unclear.

Cryo-EM structures of lambda N-TAC and *E. coli rrn*TAC at 3.7 and 4.0 Å resolution, respectively, have shed new light on the mechanisms of antitermination (39,40). In both structures, the modifying proteins interact with one another, encircling the pore of the RNA exit channel. NusB heterodimerizes with NusE(S10) and interacts specifically with *boxA* of the nascent RNA. NusG adopts an extended conformation, with its N-terminal domain binding near the active site cleft of RNAP and its C-terminal domain binding NusE(S10). In *rrn*TAC, the SuhB dimer acts as a structural hub, interacting with NusA, NusE(S10), and NusG. While unrelated and structurally dissimilar to SuhB, N plays an analogous role in the case of N-TAC, interacting with both NusA and NusE(S10). In both complexes, NusA and NusG are stabilized in ways predicted to prevent RNAP pausing and inhibit backtracking, keeping RNAP in an elongation-competent state. The bound antitermination factors probably prevent Rho-dependent and intrinsic termination by blocking Rho and NusA, respectively, from accessing the RNA exit channel.

In the early 1990s, Wagner and coworkers characterized numerous deletion and point mutations in the leader region of *rrnB* (41–43). Several mutations were found to reduce the 30S/50S ratio, 16S/23S ratio, growth rate, and activity of isolated subunits, implicating various portions of the leader in 30S subunit biogenesis. One caveat to these experiments is that they involved overexpression of rRNA to a high level (∼70% of the total rRNA), which on its own alters cell physiology. Their control strain grew with a doubling time of 40 minutes, nearly twice that of wild-type *E. coli*, and contained elevated levels of immature and/or misassembled ribosomes. Strains carrying particularly deleterious alleles of *rrnB* grew more poorly and gave rise to suppressors at high frequency. These issues, openly acknowledged by the authors, make interpretation of the reported data difficult. While the work of Wagner established the general importance of the leader RNA in 30S biogenesis, questions about the relative contributions of specific regions and features remain open.

In this work, we use an orthologous ribosome system to dissect the key functional elements of the leader and spacer regions. This system enables translation activity to be directly measured, over a >1000-fold range, without affecting cell growth (44,45). Various indicator strains, with specific codon changes in *lacZ*, allow the translational fidelity of ribosomes to be quantified in this system as well. Thus, we can obtain both quantitative (overall translation activity) and qualitative (translational accuracy) measurements of the product ribosomes, without worrying about complicating effects on cell physiology. We find that a leader-trailer helix of >17 basepairs (bp) is critical of formation of active 30S subunits in the cell. Other features, including *boxA* and leader helices hA and hB, are not strictly required. However, ribosomes formed in the absence of these elements are error-prone, suggesting that the antitermination complex and various intergenic RNA features contribute to the process and ensure quality control.

Note that throughout this article, we use the term *subunit biogenesis* to refer to the entire process of creating translationally-competent particles, including rRNA transcription.

## MATERIALS AND METHODS

### Covariation analysis

**Figure 1.**
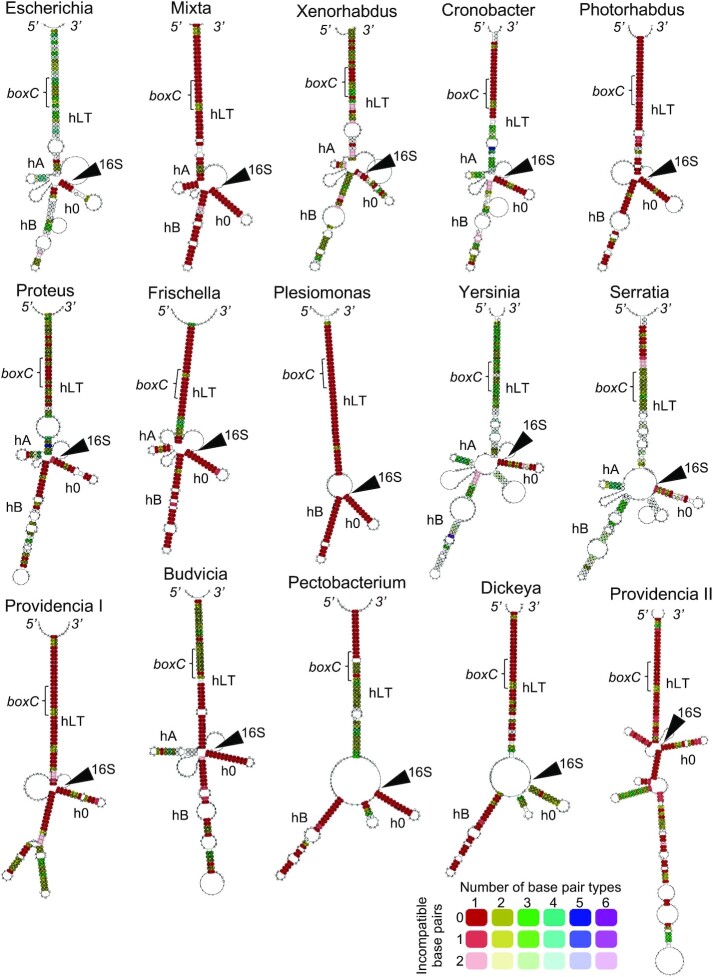
Predicted secondary structures of pre-16S rRNA in the Enterobacteriaceae. LocARNA was used to align 1441 sequences and generate a phylogenetic tree. Sequences from each of 15 clades were then subjected to co-variation analysis, resulting in the models shown. Color-coding indicates the number of different basepairs observed at a given position: red, 1; yellow, 2; green, 3; cyan, 4; blue, 5; magenta, 6. Tint level reflects the number of mismatches, with white indicating three or more mismatches. Larger high-resolution images of these models are provided in Figures S4-S18.

The genome assemblies and annotations for each of the 508 GTDB (46–49) members of the Enterobacteriaceae family listed in GTDB as NCBI type material (Supplementary Table S1) were downloaded from NCBI. For each annotated 16S rRNA, first the trailer sequence was identified by locating the conserved sequence AAGUCGUAACAAGGUA from the vicinity of the 3′ end of the mature 16S rRNA within the range from 100 nt upstream to 200 nt downstream of the annotated 16S rRNA 3′ end and then retaining the sequence from position 51 to position 107 downstream of this match. Then, the 5′ leader sequence was identified by searching for *boxC* (UCUGUGUGGG; with up to two mismatches) in the range from 257 nt to 90 nt upstream of the annotated 5′ end of the 16S rRNA and keeping the 3′ most match if multiple matches to *boxC* are found. The leader is then defined as the sequence from 23 nt upstream of the *boxC* motif to the 18th nucleotide of the annotated 16S rRNA. Sequences for which the total length of leader and trailer together was below 350 nt were discarded resulting in 1441 total rRNA leader/trailer pairs (Supplementary Table S1). The leaders and trailers were concatenated with NNNNNN representing the position of the mature 16S rRNA. Clustering of all 1441 sequences was performed via RNAclust with default parameters. The resulting tree was visualized (Supplementary Figure S1) using iTOL (50). After manual inspection of the tree, it was divided into the 15 groups shown in Figure 1 (group assignments in Supplementary Table S1). Structural alignments and conservation analysis of each group was performed using mlocarna (51–53) with default parameters.

### Orthogonal ribosome system

The translation activity of orthologous ribosomes was measured as described previously (44,54,55). Plasmid pDQ207 contains the 16S rRNA gene, *rrsB*, with the alternative ASD sequence 5′-GGGAT-3′, under transcriptional control of the P_BAD_ promoter. This plasmid lacks the 23S and 5S genes; however, expression of 16S rRNA in this context has no apparent effect on growth rate or sucrose gradient sedimentation profile. The marker mutation T1451A was engineered into pDQ207 to generate pBW022. This mutation was used to track plasmid-encoded rRNA by primer extension. Mutations and deletions were constructed using QuikChange (Stratagene) or Phusion site-directed mutagenesis (New England Biolabs) (Supplementary Table S2, Supplementary Figure S2). Plasmid pBW024 (ΔT) was generated by Gibson Assembly (NEB), where the vector and a sequence encoding the hammerhead ribozyme of *Schistosoma mansoni* (56–58) were PCR amplified with overlapping ends. Stem III of the hammerhead was engineered such that cleavage occurs 2 nt downstream of the mature 16S rRNA. To construct the HH@ series of plasmids, DNA encoding the hammerhead ribozyme was inserted at position 1612 via restriction sites engineered in plasmid pBW039, a derivative of pBW022. Then Phusion site-directed mutagenesis was used to remove a given trailer segment, fusing the hammerhead to the position denoted. With this set of constructs, stem III of the hammerhead is constant, so 7 nt (5′-GGGCAUC-3′) is left, appended to the pre-rRNA, after cleavage. These plasmid-borne 16S rRNA alleles were expressed in indicator strains KLF2674 (55), KLF2672, KLF2723, KLF3361 (45) and BRW299 (see below), enabling overall translation activity and translational fidelity to be measured, as described previously (45,55,59).

Strain KLF2513 is CSH142 carrying λ(*P_ant_* -SD_5′-ATCCC-3′_-*lacZ*). Mutation Δ*rnc::cat* was moved into KLF2513 via P1 transduction, resulting in BRW298. Then, Δ(*recA-srl*)*306 srlR::Tn10* was moved from JC14604 (E. coli Genetic Stock Center) to BRW298 via P1 transduction, resulting in the strain BRW299.

### Beta-galactosidase activity measurements

Cells from an overnight culture were diluted 300-fold into 3 ml of fresh Luria broth (LB) containing ampicillin (Amp; 100 μg/ml), kanamycin (Kan; 50 μg/ml), and l-arabinose (5 mM) and grown for 4 h at 37°C. The cells were washed once in 1 ml Z buffer (100 mM sodium phosphate at pH 7.0, 10 mM KCl, 10 mM MgSO_4_), and β-galactosidase activity was measured as described (54). Specific activity was defined by the equation: 1 unit = 1000·(*A*_574_)/(OD_600_·*v*·*t*), where *A*_574_ is absorbance at 574 nm (characteristic of the product of CPRG cleavage), OD_600_ is optical density of the cell suspension used, *v* is the volume of the cell suspension used (in milliliters), and *t* is time of incubation (in minutes) at room temperature. Strain KLF2674(pBW022) was used as the WT control and KLF2674(pBAD18) was used as the vector-only control.

### Quantification of plasmid-encoded rRNA

Cells from an overnight culture were diluted 300-fold into fresh LB (25 ml) containing Amp (100 μg/ml), Kan (50 μg/ml) and l-arabinose (5 mM) and grown for 4 h at 37°C. Cells were pelleted at 4°C, resuspended in Z buffer, transferred to a 1.5 ml Eppendorf tube, and then re-pelleted. TRIzol (Invitrogen) reagent (1 ml) was added, and the pellet was resuspended by pipetting and vortex mixing (5 min) at room temperature. Chloroform was added, mixed, then the aqueous phase was transferred to a new tube. RNA was precipitated with isopropanol then pelleted. The RNA pellet was washed with 70% ethanol and dissolved in water. Relative amounts of *P*-16S rRNA were determined using poisoned primer extension, essentially as previously described (44,60). In a 20 μl reaction, primer #1456 (5′-[Cy5]-AAAGTGGTAAGCGCCCT-3′) (0.42 pmol) was incubated with RNA (5 μg) at 50°C for 10 min in AMV buffer (NEB), AMV reverse transcriptase (NEB, 3 units), ddATP, dCTP, dGTP, and dTTP (3.5 nmol each) were added, and the reaction was incubated at 42°C for 1 h. Products were resolved by denaturing 8% PAGE, gels were imaged using a Typhoon 5 (Cytiva), and data were quantified using ImageQuant (Cytiva).

### Δ7 prrn strain construction and analysis

A recombination-deficient Δ7 prrn strain was generated by moving Δ(*recA-srl*)*306 srlR::Tn10* into SQZ10 (61) via P1 transduction, resulting in strain BRW246. Mutations to the leader and trailer region of *rrsB* were introduced into plasmid p278MS2 (62) to generate variants listed in Table 1. These plasmids were transformed into SQZ10 (or BRW246), and transformants were plated on sucrose (5%) to select against the resident plasmid pHKrrnC-sacB (54). Strains were verified by plasmid purification and sequencing. For growth rate measurements, overnight cultures were diluted 200-fold into fresh LB Amp, and growth was monitored at 37°C by measuring OD_600_ as a function of time.

**Table 1.** Ability of various *rrnB* alleles to support growth of Δ7 prrn

			Doubling time^b^	
Plasmid	Allele	Supports growth^a^	Rec^+^	Rec^−^
p278MS2	WT	Yes	35.1 ± 0.7	35.9 ± 0.5
pBW088	Δ*boxB*	Yes	37.5 ± 1.2	N.D.
pBW089	Δ*boxA*	Yes	36.4 ± 0.9	38.0 ± 0.4**
pBW090	Δ*boxBA*	Yes	35.4 ± 0.5	N.D.
pBW137	ΔhA	Yes	35.8 ± 0.6	36.9 ± 0.8
pBW138	ΔhB	Yes	36.0 ± 0.8	38.1 ± 0.7*
pBW133	Δ1543–1598	No	N.A.	N.A.
pBW134	Δ1570–1598	No	N.A.	N.A.
pBW135	Δ1571–1598	No	N.A.	N.A.
pBW136	Δ1575–1598	Yes	N.D.	58.6 ± 1.0***

N.A., not applicable; N.D., not determined.

^a^A test of whether the mutant allele is sufficient to support growth. Yes: The resident plasmid pHK*rrnC*-*sacB* of Δ7 strain SQZ10 was successfully replaced by pBWXXX. No: The frequency of sucrose resistant colonies in the counterselection step was >4 orders of magnitude lower than the control (p278MS2).

^b^Units of minutes. Data represents mean ± SEM of at least three biological replicates. Strains containing (Rec^+^) or lacking (Rec^−^) RecA were analyzed.

A two-tailed t test was used to evaluate differences from the WT: *P < 0.05; **P < 0.005; ***P < 0.0005.

Variants of strain BRW246 (WT, Δ*boxA*, Δ1575–1598, ΔhA and ΔhB) were grown to mid-log phase and subjected to sucrose gradient sedimentation analysis as described (63). Fractions (0.5 ml) were collected across the gradient, and RNA from the pre-30S, 30S and 70S regions of the gradient was extracted and analyzed by denaturing PAGE as previously described (14,64). Gels were stained with SYBR-Gold Nucleic acid stain (Invitrogen) and scanned using a Typhoon 5 (Cytiva). Data were quantified using ImageQuant (Cytiva).

## RESULTS

### Secondary structures of pre-16S rRNA in the Enterobacteriaceae

**Figure 2.**
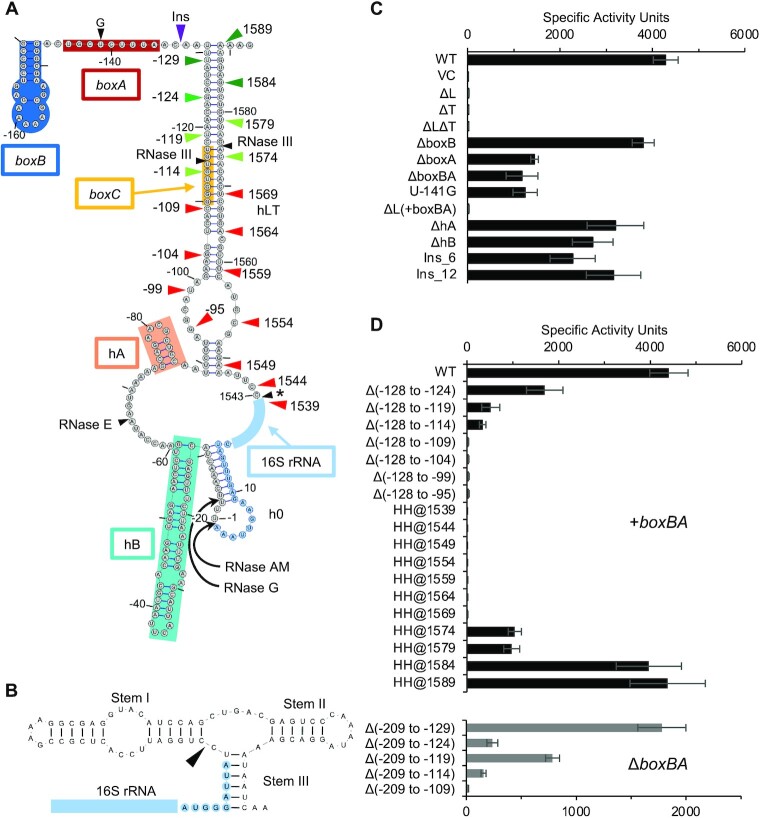
Mutagenesis of the pre-16S rRNA. (**A**) Model of the leader-trailer structure of pre-16S rRNA of *E. coli*, based on covariation analysis of the *Escherichia* clade. The location of 16S rRNA is denoted by the light blue arch, antitermination elements and helices are labeled, and RNase processing sites are denoted with black arrowheads (as indicated). The asterisk (*) denotes the site of 3′ end maturation, believed to be handled redundantly by endonuclease YbeY and several exonucleases. Green and red triangles show the endpoints of various leader or trailer deletions: dark green, full activity; light green, partial activity; red, no activity. The location of the *boxA* mutation U-141G is indicated. The purple triangle indicates the site of insertion mutations Ins_6 and Ins_12. (**B**) For constructs ΔT and ΔLΔT, a customized hammerhead ribozyme was introduced at the 3′ end of the 16S rRNA to direct efficient site-specific processing. The 5′ strand of stem III corresponds to the 3′ end of the 16S rRNA, such that cleavage (black triangle) occurs within 2 nucleotides of the natural maturation site. Light blue indicates 16S rRNA sequence, with the alternative ASD GGGAU. (**C, D**) Translation activities of ribosomes made from control and various mutant constructs (as indicated). Data represent the mean ± SEM from four or more biological replicates. VC, vector control.

Young and Steitz (1978) proposed the first secondary structure model of the pre-16S rRNA (9), which includes a long (44 bp) leader-trailer helix interrupted by two adjacent ∼10 bp leader helices (Supplementary Figure S3A). Ten years later, Schlessinger and co-workers (12) proposed a similar model, with a continuous leader-trailer helix of 30 bp and two leader helices of 4 and 19 bp (Supplementary Figure S3B). To evaluate these models, we applied covariation analysis to 1441 sequences of the family Enterobacteriaceae. Unlike the 16S rRNA itself, which is highly conserved, the flanking regions are quite variable. The RNAclust software based on LocARNA (51–53), which uses both primary sequence alignments and RNA folding algorithms, was used to build a phylogenetic tree of these sequences (Supplementary Figure S1), which we then split into 15 different clades. Sequences of each clade were then fed into LocARNA to generate a secondary structure model, with basepairs color coded to indicate the degree of covariation (Figure 1, Supplementary Figures S4–S18). One common feature of all models is a long (25–40 bp) leader-trailer helix (hLT), well supported by covariation in clades *Escherichia, Xenorhabdus, Proteus, Yersinia, Serratia, Budvicia* and *Pectobacterium* (Figure 1). In the middle of the 5′ strand of hLT lies a highly-conserved sequence, *boxC*. Despite *boxC* being nearly invariant across the family (Supplementary Table S3), covariation in this portion of hLT is still observed, due to interchangeable Watson–Crick and wobble basepairs. Another conserved helix, named here h0, forms between nt −11 to −3 and nt 7–17 of the pre-30S particle (Figure 2A). During final maturation of the subunit, h0 melts and nt 9–13 repairs with nt 21–25 (65). *Xenorhabdus, Providencia and Dickeya* show substantial covariation within h0 (Figure 1, Supplementary Figures S4-S18). A short helix of the leader, termed here hA, is seen in *Escherichia, Cronobacter, Proteus, Yersinia, Serratia, and Budvicia* structures but is absent from *Photorhabdus, Plesiomonas, Providencia, Pectobacterium* and *Dickeya* structures. Another helix of the leader, hB, ranges in size from 16–22 bp and is seen in the majority of clades analyzed. Overall, the *Escherichia* structure closely resembles that of *Mixta, Xenorhabdus, Cronobacter, Proteus* and *Frischella*. Other clades show distinguishing features, such as an additional helix between h0 and hB (*Yersinia, Serratia, Pectobacterium*, and *Dickeya*). The *Escherichia* clade structure agrees with the model of Schlessinger. However, our analysis lends no support for a conservation of base pairing between nucleotides −99 to −96 and 1554–1557 of hLT or between nucleotides −1 to −2 and 7–8 of h0 across the *Escherichia* clade (Supplementary Figure S3).

### Effects of various mutations on 30S subunit activity

To functionally characterize the leader-trailer structure in *E. coli*, we used an orthogonal ribosome system described previously (44,54,55). In this system, plasmid-encoded pre-16S rRNA with an alternative anti-Shine-Dalgarno forms 30S subunits that specifically translate chromosomally-encoded *lacZ* mRNA. Deletion of the full leader [ΔL; Δ(−209 to −1)] resulted in complete loss of activity (Figure 2A,C). Removal of the full trailer (ΔT), achieved by replacing the nt 1543–1589 with an efficient self-cleaving ribozyme from *Schistosoma mansoni* (56–58) to generate the near-precise 3′ end of the 16S rRNA (Figure 2B), also resulted in complete loss of translation activity (Figure 2A–C). Cells carrying a construct with both mutations (ΔLΔT), which should generate the mature 16S rRNA, also exhibited no detectable activity (Figure 2A–C).

Next, we sought to determine which portions of the leader are critical for 30S subunit formation. A deletion of the leader including *boxB* [Δ*boxB*; Δ(−209 to −145)] had little if any effect on translation activity (Figure 2C). Deletion of *boxA* [Δ(−147 to −133)] had a larger effect, but substantial activity (∼30%) was still seen in this strain (Figure 2C), even though *boxA* is critical for formation of the *rrn*TAC complex (38). A deletion of the leader that removes both *boxB* and *boxA* [Δ*boxBA;* Δ(−209 to −133)] caused no further loss in translation activity compared to Δ*boxA* alone. Deletion of the leader strand of hLT only, while retaining *boxB* and *boxA* [ΔL(+*boxBA*); Δ(−128 to −1)], resulted in complete loss of translation activity (Figure 2C).

Previous work has shown that a single point mutation in *boxA*, U-141G, is sufficient to prevent formation of the antitermination complex (66). In our system, U-141G reduced translation by 3-fold, just like Δ*boxA*. Thus, this 3-fold drop can be attributed to loss of transcription antitermination.

Deletion of helix hA [ΔhA; Δ(−86 to −75)] reduced translation activity by ∼30%, and deletion of hB [ΔhB; Δ(−59 to −12)] reduced activity by ∼40% (Figure 2C). These data suggest that hA and hB play relatively minor roles in 30S subunit biogenesis. The fact that these elements are functionally dispensable is consistent with their variable presence in the Enterobacteriaceae (Figure 1, Supplementary Figures S4–S18).

### Effects of progressive truncations of the leader and trailer strands

To determine the minimal leader-trailer helix needed to form functional 30S subunits, we progressively deleted portions of the leader in the presence or absence of *boxBA* (Figure 2D, top and bottom graphs). Both series showed a similar trend, with incremental decreases in activity as deletions extend from −128 to −114, and complete loss of activity when deletions extend to −109 or beyond. We also progressively deleted the trailer, by fusing the hammerhead ribozyme at various downstream positions (Figure 2D, top graph). Truncations of the trailer strand to position 1589 or 1584 had no effect; truncations to 1579 or 1574 reduced activity by ∼75%; and truncations to 1569 or further resulted in complete loss of activity. Leader and trailer deletions coincide remarkably well and show that basepairs formed by nt −113 to −89 of the leader and nt 1548–1570 of the trailer are most critical (Figure 2A, D). The base of hLT is least important, as nucleotides downstream of 1584 and upstream of −124 can be removed without loss of 30S activity.

### Effects of mutations in *boxC*

**Figure 3.**
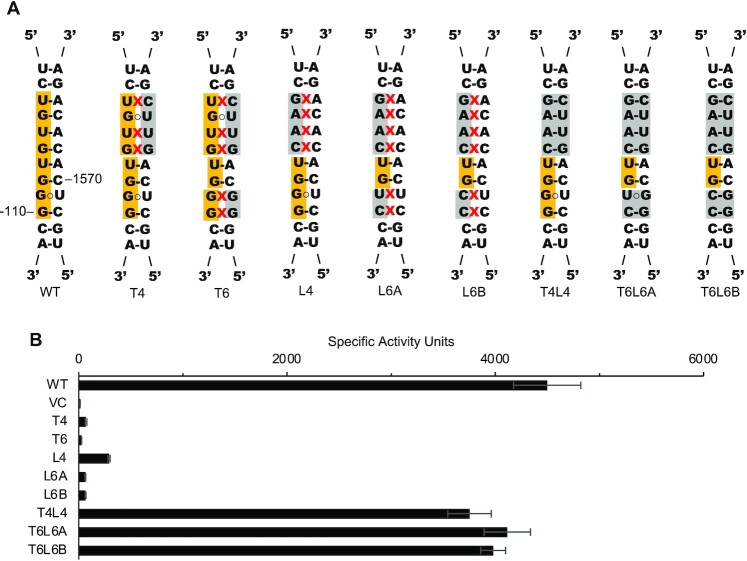
Alternative leader-trailer helices support efficient 30S subunit biogenesis. (**A**) Schematic of the *boxC* (yellow) region of hLT, showing the various mutations (grey) introduced. Mismatches are indicated with red X’s. (**B**) Translation activities of ribosomes made from constructs with mutations in the *boxC* region (as indicated). Data represent the mean ± SEM of at least four biological replicates. VC, vector control.

The sequence of *boxC* is highly conserved (Supplementary Table S3) yet its precise role remains elusive. We targeted *boxC* and the complementary portion of the trailer (Figure 3A). Disruption of base pairing by substitution of 4 or 6 nucleotides of the leader or trailer resulted in substantial loss of activity (15- to 100-fold) in all cases (Figure 3B). Surprisingly, mutations in the trailer strand had slightly more deleterious effects than those in the leader strand (i.e. *boxC*) (Figure 3B). In all cases, when base pairing in hLT was restored, so were levels of translation activity in the cell (Figure 3B). These data show that the structure of hLT is much more important than the sequence of *boxC*.

### Effects of increasing the distance between *boxA* and *boxC*

Based on their cryo-EM structure of *rrn*TAC, Wahl and coworkers hypothesized that positioning of *boxC* close to the exit channel of RNAP is important for 30S subunit assembly (40). According to their model, *boxC* remains stationary during transcription, anchored by *boxA* binding to NusB, and the nascent 16S rRNA loops out as it is synthesized. When the trailer emerges from the RNAP exit channel, *boxC* is poised to pair with the trailer sequence, enabling formation of hLT. In the Enterobacteriaceae, *boxC* is consistently positioned downstream of *boxA*, with 18 or 19 nucleotides between these elements in 98% of the cases (Supplementary Table S4). To directly test the importance of this spacing, we inserted 6 or 12 nt downstream of *boxA* (Figure 2A), which would be predicted to move *boxC* out of the *rrn*TAC channel and into the solvent. The 6-nt insertion (Ins_6) resulted in a 50% reduction in translation activity, while the 12-nt insertion (Ins_12) resulted in a 25% reduction (Figure 2C). These effects are somewhat smaller than that of Δ*boxA*, suggesting that *rrn*TAC function may only partially depend on *boxA-boxC* spacing.

### Effects in the absence of RNase III

The RNase III recognition site overlaps with *boxC* in hLT. The *rnc* gene is dispensable in *E. coli* (67,68), ruling out an essential role for RNase III in ribosome biogenesis. However, loss of translation activity coincides with removal of the RNase III site (Figure 2A, D), raising the question of whether (or to what degree) loss of RNase III activity is responsible for these phenotypes. To address this, we regenerated the complete set of orthogonal ribosome strains in the Δ*rnc* background and re-measured translation activity. Generally lower β-galactosidase levels were seen in the Δ*rnc* strain set (Supplementary Figure S19). While the basis of this effect remains unclear, some change in our readout came as no surprise. RNase III is known to cleave multiple RNA targets besides pre-rRNA in the cell, including the mRNA encoding PNPase; consequently, widespread changes to the transcriptome are evident in Δ*rnc* cells (69). Importantly, comparison of the normalized data showed that the effects of leader/trailer mutations are remarkably similar whether RNase III is present or absent (Supplementary Figure S19). In both strain sets, effects of progressive truncations of either the leader or trailer strand predicted the same critical portion of hLT. Interestingly, Δ*rnc* appeared to suppress the effects of certain leader or trailer mutations, reminiscent of data reported previously for pre-23S rRNA (70). Collectively, these observations suggest that the leader-trailer helix itself plays a critical role for 30S biogenesis, independent of RNase III.

### Effects of leader and trailer mutations on translation fidelity

**Figure 4.**
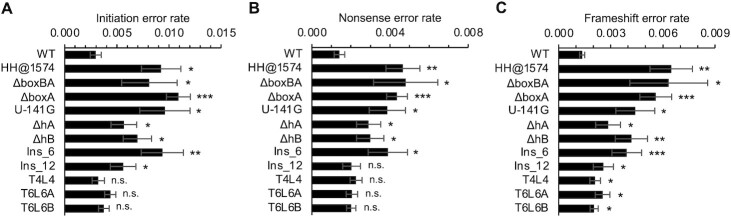
Mutations in leader and trailer result in error-prone subunits. Rates of spurious initiation from AUC (**A**), readthrough of UGA (**B**) and +1 frameshifting (**C**) of ribosomes made from various constructs (as indicated) were determined. Data represent the quotient of two means ± standard error from three or more independent biological replicates. A two-tailed *t* test was used to evaluate differences from the WT: *P < 0.05; **P < 0.005; ***P < 0.0005. n.s., not significant.

A growing body of evidence suggests that defects in 30S assembly can result in error-prone ribosomes in the translationally active pool (71–76). To investigate the effects of leader/trailer mutations on the fidelity of the subunits produced, we moved a subset of our constructs into indicator strains KLF2672, KLF2723 and KLF3361 (45). These strains are isogenic to KLF2674 (used above) but carry specific mutations in *lacZ*, enabling errors in start codon selection, decoding, and frame maintenance to be quantified. Leader mutations Δ*boxBA*, Δ*boxA*, U-141G, ΔhA, ΔhB and Ins_6 each caused significant defects in initiation and elongation fidelity (Figure 4). These mutations increased AUC initiation by 2- to 4-fold, UGA readthrough by 2-to 4-fold, and + 1 frameshifting by 2- to 5-fold. Mutation Ins_12 also conferred defects in start codon selection and frame maintenance, increasing error rates by ∼2-fold (Figure 4A, C). The trailer mutation HH@1574 also caused fidelity defects, increasing error rates in all three indicator strains by 3- to 5-fold (Figure 4). Mutations that alter the primary sequence of hLT (T4L4, T6AL6A, and T6BL6B) increased frameshift errors modestly but had no significant impact on AUC initiation or UGA readthrough (Figure 4A, B). These data suggest that intergenic RNA elements and the *rrn*TAC contribute not only to the efficiency of subunit biogenesis but also to the fidelity of the subunits produced.

### Levels of rRNA in the trailer mutant strains

**Figure 5.**
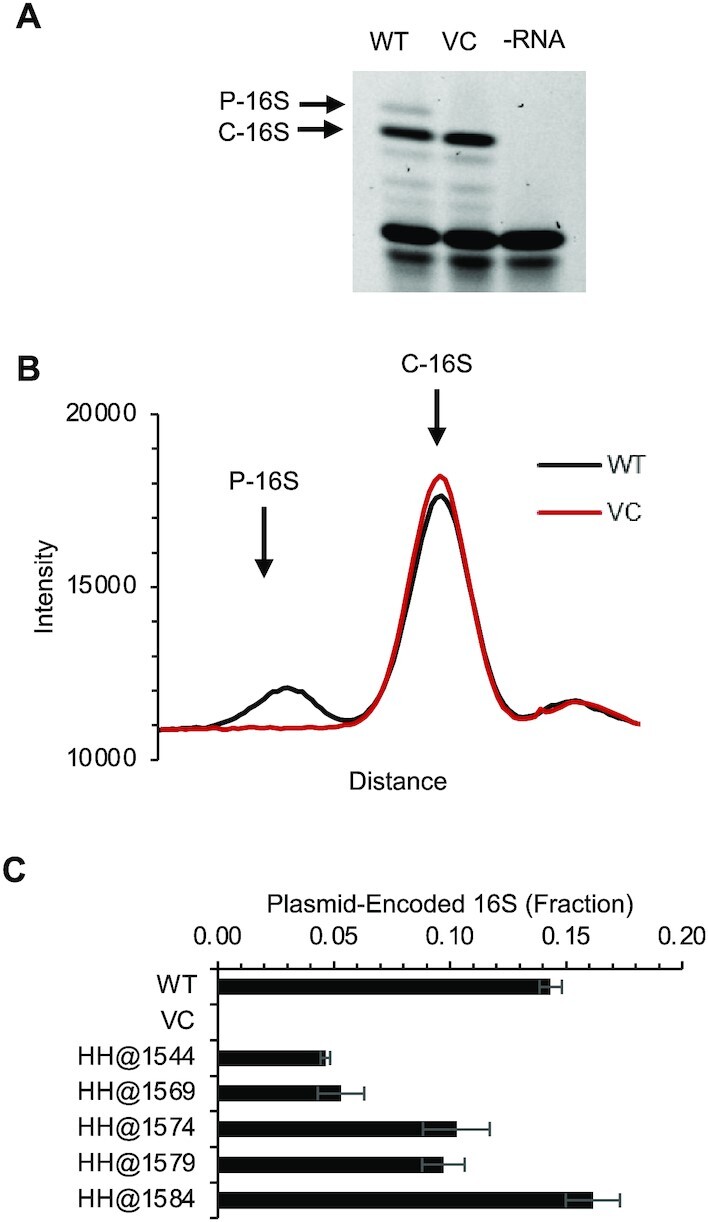
Quantification of plasmid-encoded 16S rRNA in representative strains. (**A**, **B**) An example of a poisoned primer extension experiment, showing products corresponding to plasmid-encoded (*P*-16S) and chromosomally-encoded (C-16S) rRNA. WT, wild type control; VC, vector control; -RNA, no RNA control. Panel B shows the intensity of signal along the lane for the wild type (WT) and vector control (VC) cases. (**C**) The fraction of plasmid-encoded 16S rRNA for various strains (as indicated). Data represent the mean ± SEM of at least three biological replicates.

To further explore the consequences of these mutations, we used primer extension in the presence of ddATP to quantify the levels of plasmid-encoded rRNA in various KLF2674 transformants. Strains which produced control (WT) orthogonal ribosomes exhibited ∼15% plasmid-encoded rRNA (Figure 5), in line with previous work (44). Strains carrying mutations HH@1579 or HH@1574, which produce 75% fewer active ribosomes (Figure 2D), showed 30% reduced levels of rRNA (Figure 5C). Strains which had no translation activity, HH@1544 and HH@1569 (Figure 2D), had 70% reduced levels of rRNA (Figure 5C). All these mutations lie well downstream of the promoter and are unlikely to influence transcription initiation. We envisage that these mutations confer assembly defects, and the misassembled particles are targeted for degradation, explaining the lower steady-state levels of 16S rRNA observed.

### Effects of various mutations on *E*.*coli* strain Δ7 prrn

To further evaluate the role of the *rrn*TAC on ribosomal subunit biogenesis, we moved the Δ*boxB*, Δ*boxA* and Δ*boxBA* mutations into the Δ7 prrn strain, which lacks chromosomal rRNA operons and is supported by a single plasmid-borne *rrn* operon (61,77). Deletion of *boxB* and/or *boxA* had no significant effect on the strain's doubling time (Table 1). We also moved ΔhA and ΔhB into Δ7 prrn and again, no obvious change in doubling time was seen. These data provide further evidence that these elements are dispensable for the assembly of 30S subunits.

Next, we tried to move various deletions of the trailer region into Δ7 prrn. Only the smallest deletion tested, Δ1575–1598 could be introduced (Table 1), and the resulting strain grew slowly, forming very small colonies on solid media. Attempts to measure doubling were hampered by suppressors, which arose at high frequency. We suspected that these suppressors might stem from recombination events between the introduced plasmid and ptRNA67 (77), which contains a portion of *rrnB* including the pre-16S trailer. So, we moved Δ(*recA-srl*)*306 srlR::Tn10* into SQZ10, making a recombination-deficient (Rec^−^) Δ7 prrn strain, and then replaced the resident plasmid (pHK*rrnC-sacB*) with each of several *rrnB*-containing plasmids. In this Rec^−^ background, pBW136 (Δ1575–1598) supported slow growth, and there was no indication of suppressor mutations. This enabled us to measure its doubling time, which was 23 min longer than the control strain (Table 1). Rec^−^ Δ7 prrn strains harboring Δ*boxA*, ΔhB or ΔhA grew nearly as fast as the control, with slightly higher doubling times measured in the former two cases (Table 1).

To further evaluate these mutations, we subjected the Rec^−^ Δ7 prrn strains to sucrose gradient sedimentation analysis (Supplementary Figure S20). In all cases, including the WT control, subunit peaks were considerably larger than polysome peaks, in contrast to typical profiles from wild-type *E. coli* cells (78). This suggests that ribosome biogenesis or its regulation is compromised in Δ7 prrn, which may help explain its reduced growth rate (1.7 doublings/hour) compared to wild-type *E. coli* (2.4 doublings/h) (78). None of the mutations conferred a significant change in the proportion of subunits in the context of Δ7 prrn, although the mean values for Δ1575–1598 and ΔhB are suggestive of small increases (Supplementary Figure S20B). RNA was extracted from the sucrose gradient fractions and analyzed by PAGE to evaluate pre-16S processing (Supplementary Figure S21). An RNA shorter than 16S rRNA is observed in several of the mutant strains, being most prominent in Δ7 prrn (Δ1575–1598). This product is reminiscent of 16S*, a truncated form of 16S rRNA, seen in *ΔrsgA* and *ΔrimM* strains, which are defective in 30S assembly (14,64,79). This 16S* band may stem from an error in pre-rRNA processing or reflect the initial degradation of dysfunctional subunits. Either way, the presence of 16S* in these strains provides further evidence that 30S biogenesis is negatively impacted by these mutations.

## DISCUSSION

In this work, we find that a leader-trailer helix of 17 or more basepairs is necessary for generating active 30S subunits in *E. coli*. The fact that absolutely no functional subunits are detected in the absence of hLT came as a surprise, because mature 16S rRNA supports efficient assembly of subunits *in vitro*. How can this apparent discrepancy be explained? A growing body of evidence suggests that the free energy landscape of RNA folding in the cell differs from that in the test tube (80,81). Molecular crowding, osmolytes, liquid-liquid phase condensates, and various proteins and enzymes distinguish the cytoplasmic environment from that of standard reconstitution reactions, and all these parameters impact RNA folding and dynamics. Global chemical probing studies have revealed that RNA molecules tend to be considerably less structured *in vivo* than *in vitro* (82,83). Depletion of intracellular ATP leads to increased RNA structure, suggesting that enzymes such as helicases continually promote unfolding events in the cell (83). Other recent work has shown that binding of Pumilio (PUM1/PUM2) protein to its single-stranded RNA targets is predictably inhibited by RNA structure *in vitro* but not *in vivo*, further evidence that mechanisms and/or conditions in the cell promote RNA unfolding (84). We envisage that hLT limits the conformational dynamics of pre-16S rRNA molecule by connecting its 5′ and 3′ ends. This may effectively counter the cellular drivers of unfolding and open favorable routes of 30S subunit formation.

Hammerhead cleavage leaves a 5′ hydroxyl on the ribozyme and a 2′,3′-cyclic phosphate on the upstream RNA product. This raises the question of whether this phosphate (2′,3′- or 3′-linked) might interfere with subsequent 16S rRNA processing in our system. Five enzymes have been implicated in 3′ end maturation: an endonuclease, YbeY, and four 3′-to-5′ exonucleases—RNase II, RNase R, RNase PH, and PNPase. These exonucleases act in a redundant fashion to ensure that the 3′ tail of 16S rRNA is fully trimmed (85). The activity of RNase PH on pre-tRNA substrates is strongly inhibited by a 3′ phosphate group (86), and the homologous PNPase may be similarly susceptible to such inhibition. By contrast, RNase II and RNase R are largely unaffected by the presence of a 3′- or 2′-3′-phosphate (87,88). Of the four exonucleases, RNase II and RNase R contribute most to 16S rRNA maturation, based on analysis of various triple mutant strains (85). In fact, either RNase II or RNase R is sufficient for 16S rRNA maturation (in cells containing YbeY). Given this insight, we consider it highly unlikely that hammerhead cleavage itself contributes to any substantial way to the observed effects on subunit activity. Consistent with this view, the relative production of active subunits from constructs HH@1589, HH@1584, or HH@1579 is just as high in the absence of RNase III as in its presence (Supplementary Figure S19).

In the cell, there exist pathways to target and degrade defective or damaged ribosomes. These mechanisms, while poorly understood, act on long-lived assembly intermediates and off-pathway (misassembled) particles (89–91). In the absence of hLT, slow or defective 30S biogenesis may lead to rapid degradation of 16S rRNA in assembly intermediates, further hampering production of active subunits. Indeed, we see reduced levels of plasmid-encoded rRNA when hLT cannot form, in line with active rRNA turnover. Notably, rRNA from constructs HH@1544 and HH@1569 is present at ∼30% the control level and yet no translation activity is observed, indicating some major problem in subunit assembly. Whether detectable levels of active subunits could form without the leader-trailer helix in cells lacking one or more RNases implicated in quality control remains an open question, one worth pursuing in future studies.

A growing body of evidence indicates that defects in 30S biogenesis can give rise to error-prone ribosomes. Mutation or loss of particular ribosomal proteins (*rpsE-G28E, ΔrpsO*), assembly factors (*ΔrimM, ΔksgA, ΔrbfA, ΔlepA*), or endonucleases (*Δrng, ΔybeY*) leads to increased rates of stop codon read-through, frameshifting, and/or spurious initiation (71–76). Compelling evidence suggests that quality control mechanisms are compromised in these strains, enabling premature or defective 30S particles to enter the translationally active pool. For example, Culver and coworkers found that, in strains defective in 30S assembly, precursor 17S rRNA is present in 70S ribosomes, and the level of 17S coincides with error rate (74,75). Moreover, Sharma and Anand showed that 30S subunits purified from ribosomes of *ΔksgA* or *ΔrbfA* cells have low affinity for IF3, indicating that both KsgA and RbfA are needed to enable and/or ensure IF3’s functionality on the subunit (76). Here, we show that deletion of either leader helix (ΔhA or ΔhB) or shortening of hLT by 15 bp (HH@1574) leads to reduced initiation and elongation fidelity. These data suggest that precursor RNA structures and/or dynamics also contribute to quality control. In line with this view, mutations *Δrng* and *ΔybeY* alter leader and trailer strand processing, respectively, and both mutations lead to reduced translational fidelity (71,74). How precursor RNA elements help ensure quality control remains an open question. They may for example facilitate AF action, rRNA folding, and/or rRNA processing. In general, mechanisms of quality control in bacterial ribosome biogenesis remain poorly defined. Future analyses of the mutations described here may shed light on these mechanisms.

Previous studies have shown that *boxA* is the key *cis-*acting RNA element, both necessary and sufficient for *rrn*TAC formation (22,31,92). Here, we show that deletion or mutation of *boxA* reduces 30S activity by about 3-fold, indicating that transcription antitermination moderately enhances the production of 30S subunits, in line with earlier work (66). Additionally, we find that these *boxA* mutations lead to defects in translational fidelity. This implies that *rrn*TAC not only increases transcriptional processivity (66) but also contributes in some way to the quality control of ribosome biogenesis. How *rrn*TAC acts in this regard remains unclear. It has been proposed that *rrn*TAC has RNA chaperone activity and can facilitate the folding of nascent rRNA (40). Mutations Ins_6 and Ins_12 phenocopy mutations Δ*boxA* and U-141G, which lends general support to the idea that tethering of *boxA* to NusB may indeed promote hLT formation (40). It is also possible that the role of *rrn*TAC is less direct. For example, transcription elongation speed and/or cadence may be optimal for ribosome assembly in the presence of *rrn*TAC, and suboptimal in its absence. Further studies will be needed to understand the functional link between the *rrn*TAC and the fidelity of the product ribosomes.

Like that of 16S rRNA, the precursor of 23S rRNA is bookended by a long leader-trailer helix. In *E. coli*, this 27 bp helix is processed by RNase III, RNase T, RNase AM and other RNases during 50S maturation (93), leaving the 8 bp helix H1 behind. Helix H1 lies on the solvent side of the subunit, well away from active centers of the ribosome. In some bacteria, which naturally lack H98, H1 is fully removed during 50S maturation (94). These observations suggest that the basepairs of H1 function solely in 50S biogenesis, as part of the long leader-trailer helix. Using the erythromycin-resistance mutation A1067U to distinguish plasmid- and chromosomally-encoded rRNA, Liiv and Remme performed a mutational analysis of the leader and trailer region of pre-23S rRNA in *E. coli* (70). They found that base substitutions or deletions that disrupt the leader-trailer helix have serious consequences on 50S biogenesis, akin to our current results for pre-16S rRNA. One caveat to the Liiv and Remme study is that their constructs, which carry deletions within the leader or trailer region, express pre-23S rRNA molecules that still contain 5′ and 3′ flanking RNA. These flanking sequences, which vary by construct, can potentially interact, complicating interpretation of the data. It may be worthwhile to re-visit mutagenesis of pre-23S rRNA, using a promoter and the hammerhead ribozyme to generate defined 5′ and 3′ ends, and compare the minimal pairing free energy requirements for the leader-trailer helices of pre-16S versus pre-23S particles.

Finally, we also present secondary-structure models for 15 distinct leader-trailer structures found within the Enterobacteriaceae. In all cases, a long (>30 bp) leader-trailer helix is observed, well-supported by covariation in multiple clades. Aside from this common feature, considerable structural diversity is evident (Figure 1), even though ribosomes of these organisms are highly similar. In fact, diverse leader-trailer structures are seen even within the same organism. For example, *Providencia heimbachae* has seven *rrn* operons which encode nearly identical 16S rRNA (>99.6% identity in all pairwise comparisons). Two of these operons encode the Providencia I leader-trailer structure, characterized by three leader helices of 10–15 bp; whereas, the other operons encode the Providencia II leader-trailer structure, characterized by five leader helices of 3, 6, 8, 10 and >30 bp (Figure 1). Several other organisms (*Providencia rustigianii*, *Serratia symbiotica*, *Shigella boydii*, *Shimwellia blattae*, *Tatumella citrea*, *Xenorhabdus cabanillasii*, *Yersinia aleksiciae*, *Yokenella regensburgei*) similarly have two different leader-trailer structures represented among their *rrn* operons (Supplementary Table S1). In future studies, it will be worthwhile to address whether such distinct structures play unique or common roles in the cell.

## DATA AVAILABILITY

The custom scripts used during data analysis are available at doi://10.5281/zenodo.7589875.

## Supplementary Material

gkad316_Supplemental_Files

## References

[1] Traub P. , Nomura M. Structure and function of E. coli ribosomes. V. Reconstitution of functionally active 30S ribosomal particles from RNA and proteins. Proc. Natl. Acad. Sci. U.S.A. 1968; 59:777–784. 4868216 10.1073/pnas.59.3.777 PMC224743

[2] Nierhaus K.H. , Bordasch K., Homann H.E. Ribosomal proteins. 43. In vivo assembly of Escherichia coli ribosomal proteins. J. Mol. Biol. 1973; 74:587–597. 4580907 10.1016/0022-2836(73)90049-1

[3] Maki J.A. , Schnobrich D.J., Culver G.M. The DnaK chaperone system facilitates 30S ribosomal subunit assembly. Mol. Cell. 2002; 10:129–138. 12150913 10.1016/s1097-2765(02)00562-2

[4] Datta P.P. , Wilson D.N., Kawazoe M., Swami N.K., Kaminishi T., Sharma M.R., Booth T.M., Takemoto C., Fucini P., Yokoyama S. et al. Structural aspects of RbfA action during small ribosomal subunit assembly. Mol. Cell. 2007; 28:434–445. 17996707 10.1016/j.molcel.2007.08.026 PMC2118056

[5] Razi A. , Guarné A., Ortega J. The cryo-EM structure of YjeQ bound to the 30S subunit suggests a fidelity checkpoint function for this protein in ribosome assembly. Proc. Natl. Acad. Sci. U.S.A. 2017; 114:E3396–E3403. 28396444 10.1073/pnas.1618016114 PMC5410845

[6] Xu Z. , O’Farrell H.C., Rife J.P., Culver G.M. A conserved rRNA methyltransferase regulates ribosome biogenesis. Nat. Struct. Mol. Biol. 2008; 15:534–536. 18391965 10.1038/nsmb.1408

[7] Brosius J. , Dull T.J., Sleeter D.D., Noller H.F. Gene organization and primary structure of a ribosomal RNA operon from Escherichia coli. J. Mol. Biol. 1981; 148:107–127. 7028991 10.1016/0022-2836(81)90508-8

[8] Maeda M. , Shimada T., Ishihama A. Strength and regulation of seven rRNA promoters in *Escherichia coli*. PLoS One. 2015; 10:1–19. 10.1371/journal.pone.0144697 PMC4696680 26717514

[9] Young R.A. , Steitz J.A. Complementary sequences 1700 nucleotides apart from a ribonuclease III cleavage site in *Escherichia coli* ribosomal precursor RNA. Proc. Natl. Acad. Sci. U. S. A. 1978; 75:3593–3597. 358189 10.1073/pnas.75.8.3593 PMC392831

[10] Nikolaev N. , Silengo L., Schlessinger D. A role for ribonuclease 3 in processing of ribosomal ribonucleic acid and messenger ribonucleic acid precursors in Escherichia coli. J. Biol. Chem. 1973; 248:7967–7969. 4584343

[11] Ginsburg D. , Steitz J.A. The 30 S ribosomal precursor RNA from *Escherichia coli*. J. Biol. Chem. 1975; 250:5647–5654. 1095585

[12] King T.C. , Sirdeskmukh R., Schlessinger D Nucleolytic processing of ribonucleic acid transcripts in procaryotes. Microbiol. Rev. 1986; 50:428–451. 2432388 10.1128/mr.50.4.428-451.1986 PMC373081

[13] Gupta N. , Culver G.M. Multiple in vivo pathways for *Escherichia coli* small ribosomal subunit assembly occur on one pre-rRNA. Nat. Struct. Mol. Biol. 2014; 21:937–943. 25195050 10.1038/nsmb.2887 PMC4355579

[14] Gibbs M.R. , Moon K.M., Chen M., Balakrishnan R., Foster L.J., Fredrick K. Conserved GTPase LepA (Elongation Factor 4) functions in biogenesis of the 30S subunit of the 70S ribosome. Proc. Natl. Acad. Sci. U.S.A. 2017; 114:980–985. 28096346 10.1073/pnas.1613665114 PMC5293072

[15] Inoue K. , Alsina J., Chen J., Inouye M. Suppression of defective ribosome assembly in a rbfA deletion mutant by overexpression of Era, an essential GTPase in Escherichia coli. Mol. Microbiol. 2003; 48:1005–1016. 12753192 10.1046/j.1365-2958.2003.03475.x

[16] Li J. , Horwitz R., McCracken S., Greenblatt J. NusG, a new Escherichia coli elongation factor involved in transcriptional antitermination by the N protein of phage λ. J. Biol. Chem. 1992; 267:6012–6019. 1532577

[17] Mason S.W. , Greenblatt J. Assembly of transcription elongation complexes containing the N protein of phage λ and the *Escherichia coli* elongation factors NusA, NusB, NusG, and S10. Genes Dev. 1991; 5:1504–1512. 1831176 10.1101/gad.5.8.1504

[18] Friedman D.I. , Baron L.S. Genetic characterization of a bacterial locus involved in the activity of the N function of phage λ. Virology. 1974; 58:141–148. 4595374 10.1016/0042-6822(74)90149-4

[19] Nudler E. , Gottesman M.E. Transcription termination and anti-termination in E. coli. Genes Cells. 2002; 7:755–768. 12167155 10.1046/j.1365-2443.2002.00563.x

[20] Schärpf M. , Sticht H., Schweimer K., Boehm M., Hoffmann S., Rösch P. Antitermination in bacteriophage λ. Eur. J. Biochem. 2000; 267:2397–2408. 10759866 10.1046/j.1432-1327.2000.01251.x

[21] Legault P. , Li J., Mogridge J., Kay L.E., Greenblatt J. NMR structure of the bacteriophage λ N peptide/boxB RNA complex: recognition of a GNRA fold by an arginine-rich motif. Cell. 1998; 93:289–299. 9568720 10.1016/s0092-8674(00)81579-2

[22] Berg K.L. , Squires C., Squires C.L. Ribosomal RNA operon anti-termination. Function of leader and spacer region box B-box A sequences and their conservation in diverse micro-organisms. J. Mol. Biol. 1989; 209:345–358. 2479752 10.1016/0022-2836(89)90002-8

[23] Friedman D.I. , Schauer A.T., Baumann M.R., Baron L.S., Adhya S.L. Evidence that ribosomal protein S10 participates in control of transcription termination. Proc. Natl. Acad. Sci. U.S.A. 1981; 78:1115–1118. 6453343 10.1073/pnas.78.2.1115 PMC319957

[24] Das A. , Ghosh B., Barik S., Wolska K. Evidence that ribosomal protein S10 itself is a cellular component necessary for transcription antitermination by phage λ N protein. Proc. Natl. Acad. Sci. U.S.A. 1985; 82:4070–4074. 2987961 10.1073/pnas.82.12.4070 PMC397936

[25] Downing W.L. , Sullivan S.L., Gottesman M.E., Dennis P.P. Sequence and transcriptional pattern of the essential Escherichia coli secE-nusG operon. J. Bacteriol. 1990; 172:1621–1627. 2137819 10.1128/jb.172.3.1621-1627.1990 PMC208640

[26] Sullivan S.L. , Gottesman M.E. Requirement for *E. coli* NusG protein in factor-dependent transcription termination. Cell. 1992; 68:989–994. 1547498 10.1016/0092-8674(92)90041-a

[27] Friedman D.I. , Wilgus G.S., Mural R.J. Gene N regulator function of phage λimm21: evidence that a site of N action differs from a site of N recognition. J. Mol. Biol. 1973; 81:505–516. 4778807 10.1016/0022-2836(73)90519-6

[28] Adhya S. , Gottesman M., De Crombrugghe B. Release of polarity in *Escherichia coli* by gene N of phage λ: termination and antitermination of transcription. Proc. Natl. Acad. Sci. U.S.A. 1974; 71:2534–2538. 4601822 10.1073/pnas.71.6.2534 PMC388494

[29] Franklin N.C. Altered reading of genetic signals fused to the N operon of bacteriophage λ: genetic evidence for modification of polymerase by the protein product of the N gene. J. Mol. Biol. 1974; 89:33–48. 4613856 10.1016/0022-2836(74)90161-2

[30] Roberts J.W. Phage lambda and the regulation of transcription termination. Cell. 1988; 52:5–6. 2449971 10.1016/0092-8674(88)90523-5

[31] Vogel U. , Jensen K.F. Effects of the antiterminator boxA on transcription elongation kinetics and ppGpp inhibition of transcription elongation in *Escherichia coli*. J. Biol. Chem. 1995; 270:18335–18340. 7629155 10.1074/jbc.270.31.18335

[32] Huang Y.H. , Said N., Loll B., Wahl M.C. Structural basis for the function of SuhB as a transcription factor in ribosomal RNA synthesis. Nucleic Acids Res. 2019; 47:6488–6503. 31020314 10.1093/nar/gkz290 PMC6614801

[33] Singh N. , Bubunenko M., Smith C., Abbott D.M., Stringer A.M., Shi R., Court D.L., Wadea J.T. SuhB associates with Nus factors to facilitate 30S ribosome biogenesis in *Escherichia coli*. MBio. 2016; 7:e00114–16. 26980831 10.1128/mBio.00114-16 PMC4807359

[34] Torres M. , Condon C., Balada J.M., Squires C., Squires C.L. Ribosomal protein S4 is a transcription factor with properties remarkably similar to NusA, a protein involved in both non-ribosomal and ribosomal RNA antitermination. EMBO J. 2001; 20:3811–3820. 11447122 10.1093/emboj/20.14.3811 PMC125540

[35] Sharrock R.A. , Gourse R.L., Nomura M. Defective antitermination of rRNA transcription and derepression of rRNA and tRNA synthesis in the nusB5 mutant of Escherichia coli. Proc. Natl. Acad. Sci. U.S.A. 1985; 82:5275–5279. 3161080 10.1073/pnas.82.16.5275 PMC390550

[36] Squires C.L. , Greenblatt J., Li J., Condon C., Squires C.L. Ribosomal RNA antitermination in vitro: requirement for Nus factors and one or more unidentified cellular components. Proc. Natl. Acad. Sci. U.S.A. 1993; 90:970–974. 8430111 10.1073/pnas.90.3.970 PMC45792

[37] Morgan E.A. Antitermination mechanisms in rRNA operons of *Escherichia coli*. J. Bacteriol. 1986; 168:1–5. 2428806 10.1128/jb.168.1.1-5.1986 PMC213412

[38] Gourse R.L. , de Boer H.A., Nomura M. DNA determinants of rRNA synthesis in *E. coli*: growth rate dependent regulation, feedback inhibition, upstream activation, antitermination. Cell. 1986; 44:197–205. 2416474 10.1016/0092-8674(86)90498-8

[39] Krupp F. , Said N., Huang Y.H., Loll B., Bürger J., Mielke T., Spahn C.M.T., Wahl M.C. Structural basis for the action of an all-purpose transcription anti-termination factor. Mol. Cell. 2019; 74:143–157. 30795892 10.1016/j.molcel.2019.01.016

[40] Huang Y.H. , Hilal T., Loll B., Bürger J., Mielke T., Böttcher C., Said N., Wahl M.C. Structure-based mechanisms of a molecular RNA polymerase/chaperone machine required for ribosome biosynthesis. Mol. Cell. 2020; 79:1024–1036. 32871103 10.1016/j.molcel.2020.08.010

[41] Theißen G. , Behrens S.E., Wagner R. Functional importance of the Escherichia coli ribosomal RNA leader box A sequence for post-transcriptional events. Mol. Microbiol. 1990; 4:1667–1678. 1981803 10.1111/j.1365-2958.1990.tb00544.x

[42] Theißen G. , Eberie J., Zacharias M., Tobias L., Wagner R. The tL structure within the leader region of Escherichia coli ribosomal RNA operons has post-transcriptional functions. Nucleic Acids Res. 1990; 18:3893–3901. 2197598 10.1093/nar/18.13.3893 PMC331091

[43] Theissen G. , Thelen L., Wagner R. Some base substitutions in the leader of an Escherichia coli ribosomal RNA operon affect the structure and function of ribosomes. Evidence for a transient scaffold function of the rRNA leader. J. Mol. Biol. 1993; 233:203–218. 8377198 10.1006/jmbi.1993.1500

[44] Abdi N.M. , Fredrick K. Contribution of 16S rRNA nucleotides forming the 30S subunit A and P sites to translation in Escherichia coli. RNA. 2005; 11:1624–1632. 16177132 10.1261/rna.2118105 PMC1370848

[45] McClory S.P. , Devaraj A., Qin D., Leisring J.M., Fredrick K. Rodnina M.V. , Wintermeyer W., Green R. Chapter 19: mutations in 16S rRNA that decrease the fidelity of translation. Ribosome Structure, Function, and Dynamics. 2011; New York Springer Wien 237–247.

[46] Parks D.H. , Chuvochina M., Waite D.W., Rinke C., Skarshewski A., Chaumeil P.A., Hugenholtz P. A standardized bacterial taxonomy based on genome phylogeny substantially revises the tree of life. Nat. Biotechnol. 2018; 36:996. 30148503 10.1038/nbt.4229

[47] Parks D.H. , Chuvochina M., Chaumeil P.A., Rinke C., Mussig A.J., Hugenholtz P. A complete domain-to-species taxonomy for bacteria and archaea. Nat. Biotechnol. 2020; 38:1079–1086. 32341564 10.1038/s41587-020-0501-8

[48] Rinke C. , Chuvochina M., Mussig A.J., Chaumeil P.A., Davín A.A., Waite D.W., Whitman W.B., Parks D.H., Hugenholtz P. A standardized archaeal taxonomy for the Genome Taxonomy Database. Nat. Microbiol. 2021; 6:946–959. 34155373 10.1038/s41564-021-00918-8

[49] Parks D.H. , Chuvochina M., Rinke C., Mussig A.J., Chaumeil P.A., Hugenholtz P. GTDB: an ongoing census of bacterial and archaeal diversity through a phylogenetically consistent, rank normalized and complete genome-based taxonomy. Nucleic Acids Res. 2022; 50:D785–D794. 34520557 10.1093/nar/gkab776 PMC8728215

[50] Letunic I. , Bork P. Interactive Tree of Life (iTOL) v4: recent updates and new developments. Nucleic Acids Res. 2019; 47:256–259. 10.1093/nar/gkz239 PMC6602468 30931475

[51] Will S. , Reiche K., Hofacker I.L., Stadler P.F., Backofen R. Inferring noncoding RNA families and classes by means of genome-scale structure-based clustering. PLoS Comput. Biol. 2007; 3:680–691. 10.1371/journal.pcbi.0030065 PMC1851984 17432929

[52] Will S. , Joshi T., Hofacker I.L., Stadler P.F., Backofen R. LocARNA-P: accurate boundary prediction and improved detection of structural rnas. RNA. 2012; 18:900–914. 22450757 10.1261/rna.029041.111 PMC3334699

[53] Raden M. , Ali S.M., Alkhnbashi O.S., Busch A., Costa F., Davis J.A., Eggenhofer F., Gelhausen R., Georg J., Heyne S. et al. Freiburg RNA tools: a central online resource for RNA-focused research and teaching. Nucleic Acids Res. 2018; 46:W25–W29. 29788132 10.1093/nar/gky329 PMC6030932

[54] Qin D. , Abdi N.M., Fredrick K. Characterization of 16S rRNA mutations that decrease the fidelity of translation initiation. RNA. 2007; 13:2348–2355. 17942743 10.1261/rna.715307 PMC2080605

[55] McClory S.P. , Leisring J.M., Qin D., Fredrick K. Missense suppressor mutations in 16S rRNA reveal the importance of helices h8 and h14 in aminoacyl-tRNA selection. RNA. 2010; 16:1925–1934. 20699303 10.1261/rna.2228510 PMC2941101

[56] Ferbeyre G. , Smith J.M., Cedergren R. Schistosome satellite DNA encodes active hammerhead ribozymes. Mol. Cell. Biol. 1998; 18:3880–3888. 9632772 10.1128/mcb.18.7.3880 PMC108972

[57] Canny M.D. , Jucker F.M., Kellogg E., Khvorova A., Jayasena S.D., Pardi A. Fast cleavage kinetics of a natural hammerhead ribozyme. J. Am. Chem. Soc. 2004; 126:10848–10849. 15339162 10.1021/ja046848v

[58] Chen M. , Fredrick K. Measures of single-versus multiple-round translation argue against a mechanism to ensure coupling of transcription and translation. Proc. Natl. Acad. Sci. U. S. A. 2018; 115:10774–10779. 30275301 10.1073/pnas.1812940115 PMC6196535

[59] Qin D. , Fredrick K. Control of translation initiation involves a factor-induced rearrangement of helix 44 of 16S ribosomal RNA. Mol. Microbiol. 2009; 71:1239–1249. 19154330 10.1111/j.1365-2958.2009.06598.x PMC3647337

[60] McNutt Z.A. , Gandhi M.D., Shatoff E.A., Roy B., Devaraj A., Bundschuh R., Fredrick K. Comparative analysis of anti-Shine-Dalgarno function in Flavobacterium johnsoniae and Escherichia coli. Front. Mol. Biosci. 2021; 8:787388. 34966783 10.3389/fmolb.2021.787388 PMC8710568

[61] Quan S. , Skovgaard O., McLaughlin R.E., Buurman E.T., Squires C.L. Markerless Escherichia coli rrn deletion strains for genetic determination of ribosomal binding sites. G3 Genes Genomes Genet. 2015; 5:2555–2557. 10.1534/g3.115.022301 PMC4683628 26438293

[62] Youngman E.M. , Brunelle J.L., Kochaniak A.B., Green R. The active site of the ribosome is composed of two layers of conserved nucleotides with distinct roles in peptide bond formation and peptide release. Cell. 2004; 117:589–599. 15163407 10.1016/s0092-8674(04)00411-8

[63] Qin D. , Fredrick K. Analysis of polysomes from bacteria. Methods Enzymol. 2013; 530:159–172. 24034320 10.1016/B978-0-12-420037-1.00008-7

[64] Leong V. , Kent M., Jomaa A., Ortega J. Escherichia coli rimM and yjeQ null strains accumulate immature 30S subunits of similar structure and protein complement. Rna. 2013; 19:789–802. 23611982 10.1261/rna.037523.112 PMC3683913

[65] Dammel C.S. , Noller H.F. A cold-sensitive mutation in 16S rRNA provides evidence for helical switching in ribosome assembly. Genes Dev. 1993; 7:660–670. 7681419 10.1101/gad.7.4.660

[66] Heinrich T. , Condon C., Pfeiffer T., Hartmann R.K. Point mutations in the leader boxA of a plasmid-encoded Escherichia coli rrnB operon cause defective antitermination in vivo. J. Bacteriol. 1995; 177:3793–3800. 7601845 10.1128/jb.177.13.3793-3800.1995 PMC177098

[67] Takiff H.E. , Chen S.M., Court D.L. Genetic analysis of the rnc operon of Escherichia coli. J. Bacteriol. 1989; 171:2581–2590. 2540151 10.1128/jb.171.5.2581-2590.1989 PMC209937

[68] Naganathan A. , Keltz R., Lyon H., Culver G.M. Uncovering a delicate balance between endonuclease RNase III and ribosomal protein S15 in E. coli ribosome assembly. Biochimie. 2021; 191:104–117. 34508826 10.1016/j.biochi.2021.09.003 PMC8627457

[69] Gordon G.C. , Cameron J.C., Pfleger B.F. RNA sequencing identifies new Rnase III cleavage sites in Escherichia coli and reveals increased regulation of mRNA. MBio. 2017; 8:e00128–17. 28351917 10.1128/mBio.00128-17 PMC5371410

[70] Liiv A. , Remme J. Base-pairing of 23 S rRNA ends is essential for ribosomal large subunit assembly. J. Mol. Biol. 1998; 276:537–545. 9551095 10.1006/jmbi.1997.1532

[71] Davies B.W. , Köhrer C., Jacob A.I., Simmons L.A., Zhu J., Aleman L.M., RajBhandary U.L., Walker G.C. Role of *Escherichia coli* YbeY, a highly conserved protein, in rRNA processing. Mol. Microbiol. 2010; 78:506–518. 20807199 10.1111/j.1365-2958.2010.07351.x PMC2959132

[72] Roy-Chaudhuri B. , Kirthi N., Kelley T., Culver G.M. Suppression of a cold-sensitive mutation in ribosomal protein S5 reveals a role for RimJ in ribosome biogenesis. Mol. Microbiol. 2008; 68:1547–1559. 18466225 10.1111/j.1365-2958.2008.06252.x PMC2440530

[73] Kirthi N. , Roy-Chaudhuri B., Kelley T., Culver G.M. A novel single amino acid change in small subunit ribosomal protein S5 has profound effects on translational fidelity. RNA. 2006; 12:2080–2091. 17053085 10.1261/rna.302006 PMC1664723

[74] Roy-Chaudhuri B. , Kirthi N., Culver G.M. Appropriate maturation and folding of 16S rRNA during 30S subunit biogenesis are critical for translational fidelity. Proc. Natl. Acad. Sci. U.S.A. 2010; 107:4567–4572. 20176963 10.1073/pnas.0912305107 PMC2842029

[75] Connolly K. , Culver G. Overexpression of RbfA in the absence of the KsgA checkpoint results in impaired translation initiation. Mol. Microbiol. 2013; 87:968–981. 23387871 10.1111/mmi.12145 PMC3583373

[76] Sharma H. , Anand B. Ribosome assembly defects subvert initiation Factor3 mediated scrutiny of bona fide start signal. Nucleic Acids Res. 2019; 47:11368–11386. 31586395 10.1093/nar/gkz825 PMC6868393

[77] Asai T. , Zaporojets D., Squires C., Squires C.L. An Escherichia coli strain with all chromosomal rRNA operons inactivated: complete exchange of rRNA genes between bacteria. Proc. Natl. Acad. Sci. U.S.A. 1999; 96:1971–1976. 10051579 10.1073/pnas.96.5.1971 PMC26721

[78] Balakrishnan R. , Oman K., Shoji S., Bundschuh R., Fredrick K. The conserved GTPase LepA contributes mainly to translation initiation in *Escherichia coli*. Nucleic Acids Res. 2014; 42:13370–13383. 25378333 10.1093/nar/gku1098 PMC4245954

[79] Himeno H. , Hanawa-Suetsugu K., Kimura T., Takagi K., Sugiyama W., Shirata S., Mikami T., Odagiri F., Osanai Y., Watanabe D. et al. A novel GTPase activated by the small subunit of ribosome. Nucleic Acids Res. 2004; 32:5303–5309. 15466596 10.1093/nar/gkh861 PMC521671

[80] Ganser L.R. , Kelly M.L., Herschlag D., Al-Hashimi H.M. The roles of structural dynamics in the cellular functions of RNAs. Nat. Rev. Mol. Cell Biol. 2019; 20:474–489. 31182864 10.1038/s41580-019-0136-0 PMC7656661

[81] Leamy K.A. , Assmann S.M., Mathews D.H., Bevilacqua P.C. Bridging the gap between in vitro and in vivo RNA folding. Q. Rev. Biophys. 2016; 49:e10. 27658939 10.1017/S003358351600007X PMC5269127

[82] Mustoe A.M. , Busan S., Rice G.M., Hajdin C.E., Peterson B.K., Ruda V.M., Kubica N., Nutiu R., Baryza J.L., Weeks K.M. Pervasive regulatory functions of mRNA structure revealed by high-resolution SHAPE probing. Cell. 2018; 173:181–195. 29551268 10.1016/j.cell.2018.02.034 PMC5866243

[83] Rouskin S. , Zubradt M., Washietl S., Kellis M., Weissman J.S. Genome-wide probing of RNA structure reveals active unfolding of mRNA structures in vivo. Nature. 2014; 505:701–705. 24336214 10.1038/nature12894 PMC3966492

[84] Jarmoskaite I. , Denny S.K., Vaidyanathan P.P., Becker W.R., Andreasson J.O.L., Layton C.J., Kappel K., Shivashankar V., Sreenivasan R., Das R. et al. A quantitative and predictive model for RNA binding by Human Pumilio proteins. Mol. Cell. 2019; 74:966–981. 31078383 10.1016/j.molcel.2019.04.012 PMC6645366

[85] Sulthana S. , Deutscher M.P. Multiple exoribonucleases catalyze maturation of the 3′ terminus of 16S ribosomal RNA (rRNA). J. Biol. Chem. 2013; 288:12574–12579. 23532845 10.1074/jbc.C113.459172 PMC3642305

[86] Kelly K.O. , Deutscher M.P. Characterization of *Escherichia coli* RNase PH. J. Biol. Chem. 1992; 267:17153–17158. 1512253

[87] Cheng Z.F. , Deutscher M.P. Purification and characterization of the *Escherichia coli* exoribonuclease Rnase R. Comparison with RNase II. J. Biol. Chem. 2002; 277:21624–21629. 11948193 10.1074/jbc.M202942200

[88] Cannistraro V.J. , Kennell D The processive reaction mechanism of ribonuclease II. J. Mol. Biol. 1994; 243:930–943. 7966309 10.1006/jmbi.1994.1693

[89] Cheng Z.-F. , Deutscher M.P. Quality control of ribosomal RNA mediated by polynucleotide phosphorylase and RNase R. Proc. Natl. Acad. Sci. U.S.A. 2003; 100:6388–6393. 12743360 10.1073/pnas.1231041100 PMC164456

[90] Maiväli Ü. , Paier A., Tenson T. When stable RNA becomes unstable: the degradation of ribosomes in bacteria and beyond. Biol. Chem. 2013; 394:845–855. 23612597 10.1515/hsz-2013-0133

[91] Jain C. Role of ribosome assembly in Escherichia coli ribosomal RNA degradation. Nucleic Acids Res. 2018; 46:11048–11060. 30219894 10.1093/nar/gky808 PMC6237783

[92] Olson E.R. , Tomich C.S., Friedman D.I. The nusA recognition site. Alteration in its sequence or position relative to upstream translation interferes with the action of the N antitermination function of phage lambda. J. Mol. Biol. 1984; 180:1053–1063. 6098688 10.1016/0022-2836(84)90270-5

[93] Jain C. RNase AM, a 5′ to 3′ exonuclease, matures the 5′ end of all three ribosomal RNAs in *E. coli*. Nucleic Acids Res. 2020; 48:5616–5623. 32343306 10.1093/nar/gkaa260 PMC7261194

[94] Shatoff E.A. , Gemler B.T., Bundschuh R., Fredrick K. Maturation of 23S rRNA includes removal of helix H1 in many bacteria. RNA Biol. 2021; 18:856–865. 34812116 10.1080/15476286.2021.2000793 PMC8782170

