# Supplementary material for: Roles of the leader-trailer helix and antitermination complex in biogenesis of the 30S ribosomal subunit
Source: Nucleic Acids Res. 2023 Apr 27;51(10):5242–54. doi: 10.1093/nar/gkad316 (PMC10250234; doi:10.1093/nar/gkad316)
Supplement: gkad316_Supplemental_Files [file gkad316_supplemental_files.zip › Fig_S2.pdf]

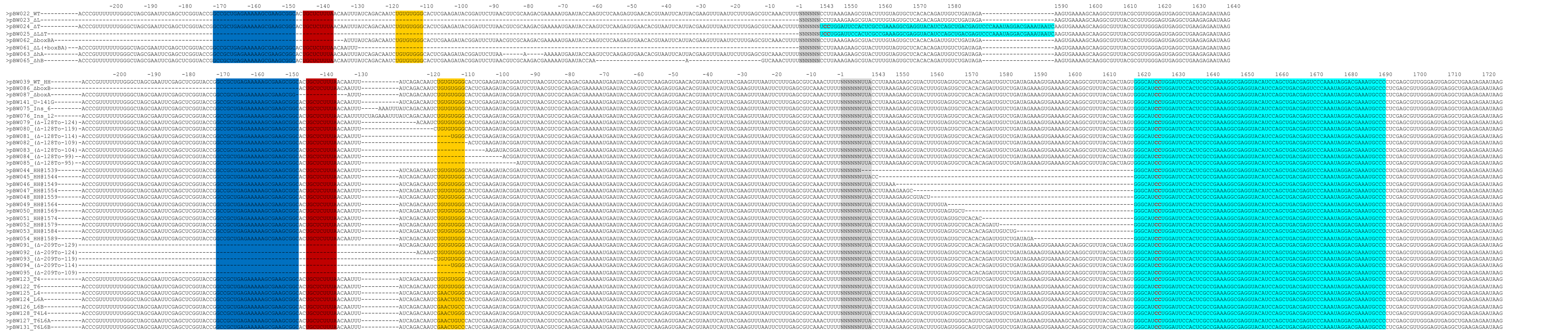

Figure S2. Sequences of pre-16S rRNA produced from constructs used in this study. Plasmid name and construct nomenclature followed by the sequence starting at the first nucleotide transcribed. The top set of correspond to derivatives of pBW022; the bottom set correspond to derivatives of pBW039. "NNNNNN" denotes the location of the 16S rRNA. Color code: blue, *boxB*; red, *boxA*; gold, *boxC*; grey, 16S rRNA; teal, hammerhead ribozyme. Hammerhead cleavage occurs between the two cytidines highlighted in red bold font.
