# Supplementary material for: Roles of the leader-trailer helix and antitermination complex in biogenesis of the 30S ribosomal subunit
Source: Nucleic Acids Res. 2023 Apr 27;51(10):5242–54. doi: 10.1093/nar/gkad316 (PMC10250234; doi:10.1093/nar/gkad316)
Supplement: gkad316_Supplemental_Files [file gkad316_supplemental_files.zip › Legend_Fig_S1.docx]

**Figure S1**. Phylogenetic tree based on sequence and structural similarity of 1441 Enterobacteriaceae leader-trailer sequences constructed with the RNAclust tool. This tree forms the basis of the selection of the 15 clades, the structural models of which are shown in Figure 1.
