# Supplementary material for: Roles of the leader-trailer helix and antitermination complex in biogenesis of the 30S ribosomal subunit
Source: Nucleic Acids Res. 2023 Apr 27;51(10):5242–54. doi: 10.1093/nar/gkad316 (PMC10250234; doi:10.1093/nar/gkad316)
Supplement: gkad316_Supplemental_Files [file gkad316_supplemental_files.zip › SUP-R1.pdf]

Supplementary Data for

Roles of the leader-trailer helix and antitermination complex in biogenesis of the 30S ribosomal subunit

Benjamin R. Warner[1,2], Ralf Bundschuh[2,3,4,5] and Kurt Fredrick[1,2]*

[1]Department of Microbiology, The Ohio State University, Columbus, Ohio 43210, USA

[2]Center for RNA Biology, The Ohio State University, Columbus, Ohio 43210, USA

[3]Department of Physics, The Ohio State University, Columbus, Ohio 43210, USA

[4]Department of Chemistry and Biochemistry, The Ohio State University, Columbus, Ohio 43210, USA

[5]Division of Hematology, Department of Internal Medicine, The Ohio State University, Columbus Ohio 43210, USA

This supplement contains:

Figures S3 to S21

Tables S2 to S4

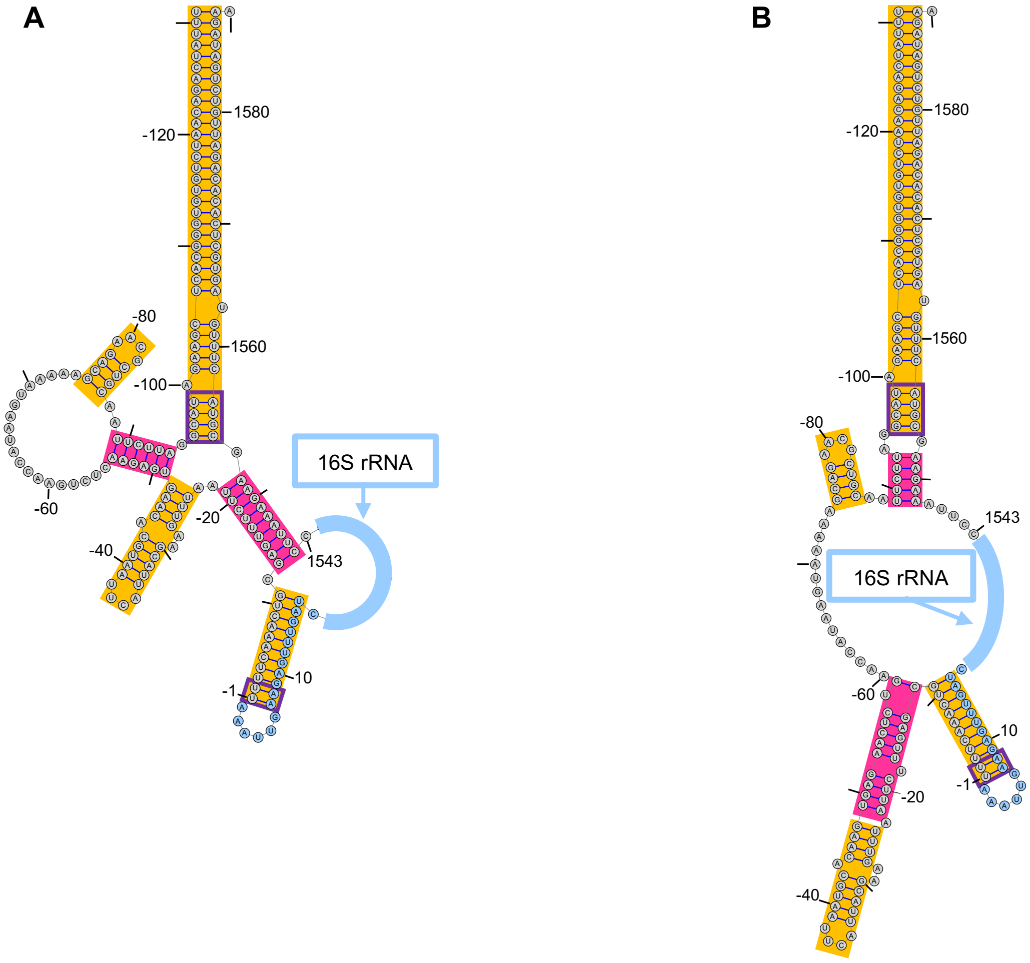

Figure S3. Comparison of different leader-trailer models. (A) Model of Young and Steitz (1978). (B) Model of Schlessinger and coworkers (1986). Orange boxes represent agreement between models, whereas magenta boxes represent disagreement. Purple boxes highlight based pairs that are non-conserved across the *Escherichia* clade (this work).

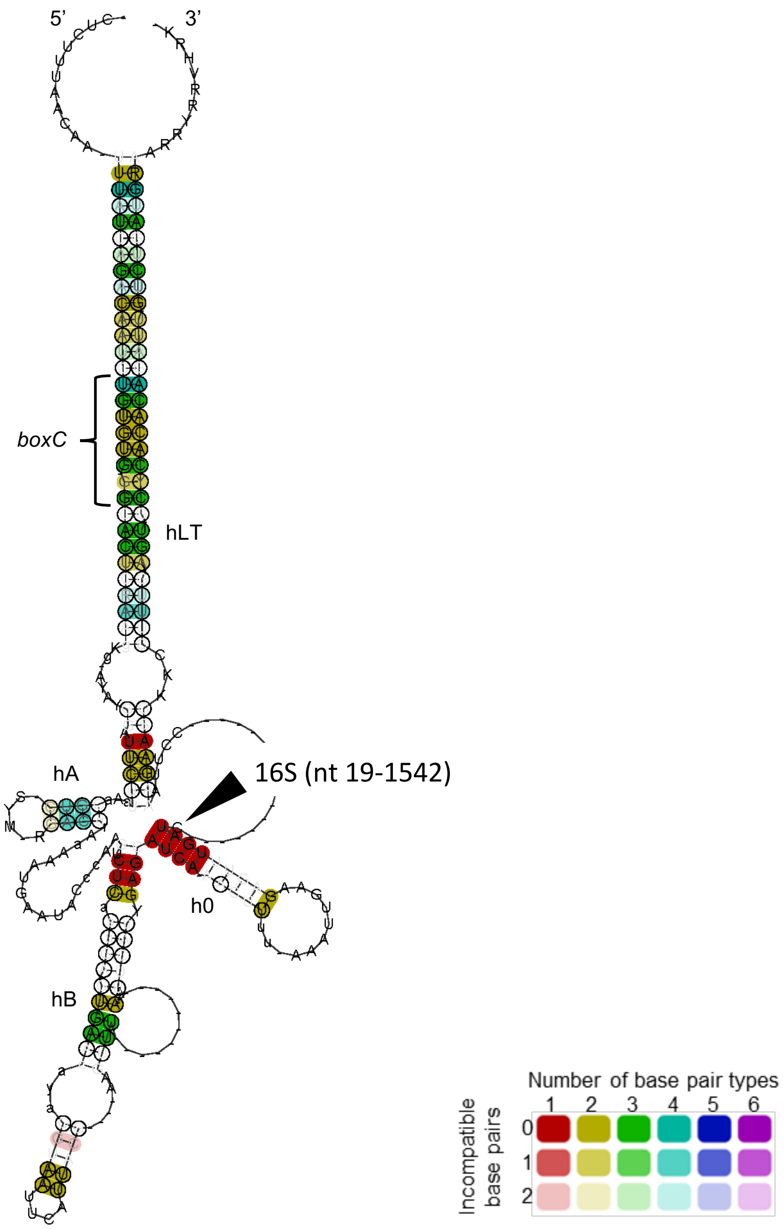

Figure S4. Secondary structure derived for the *Escherichia* clade (node: N627, n=628) of Enterobacteriaceae.

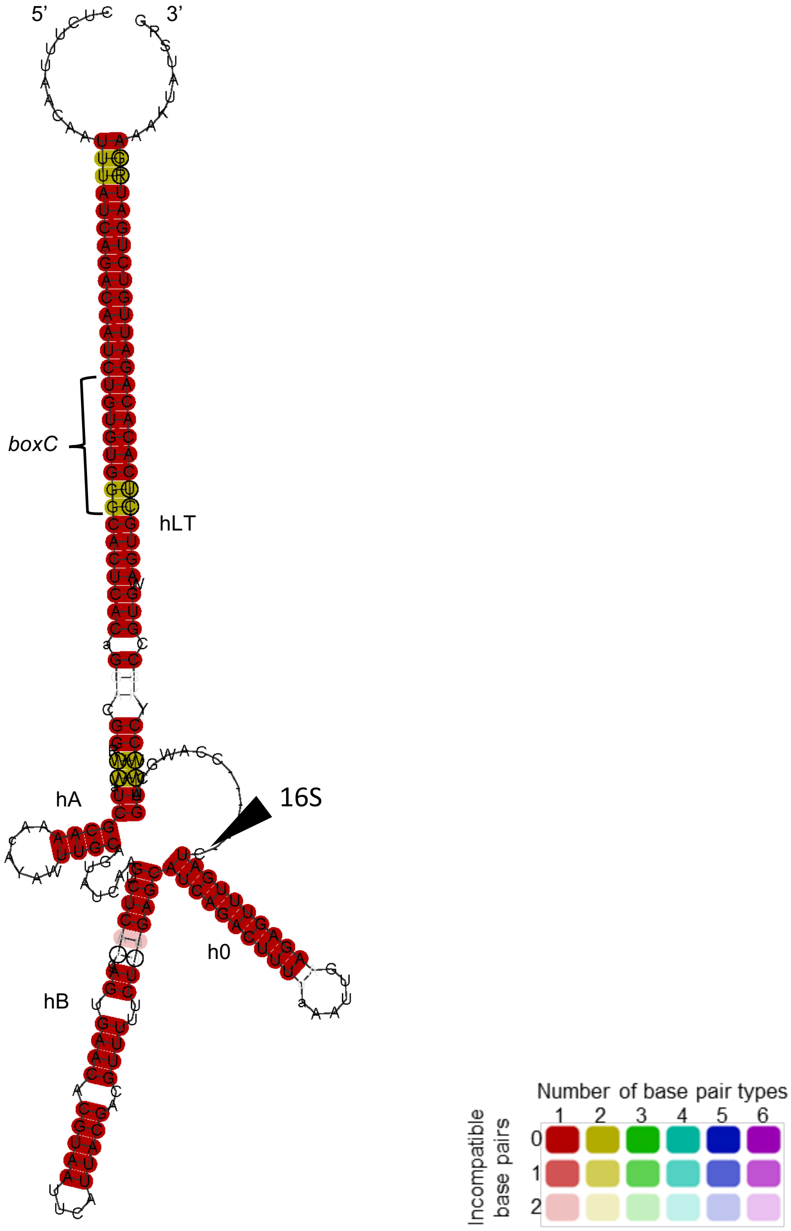

Figure S5. Secondary structure derived for the *Mixta* clade (node: N647, n=21) of Enterobacteriaceae.

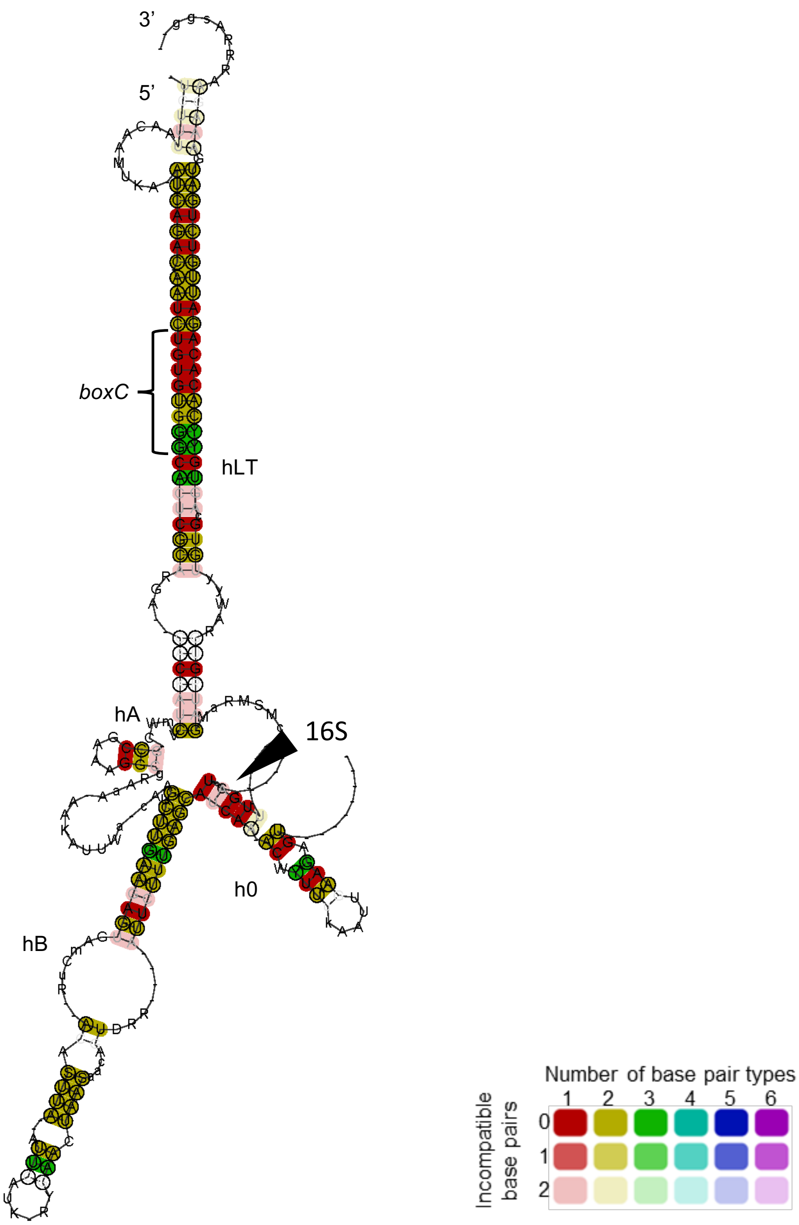

Figure S6. Secondary structure derived for the *Xenorhabdus* clade (node: N688, n=41) of Enterobacteriaceae.

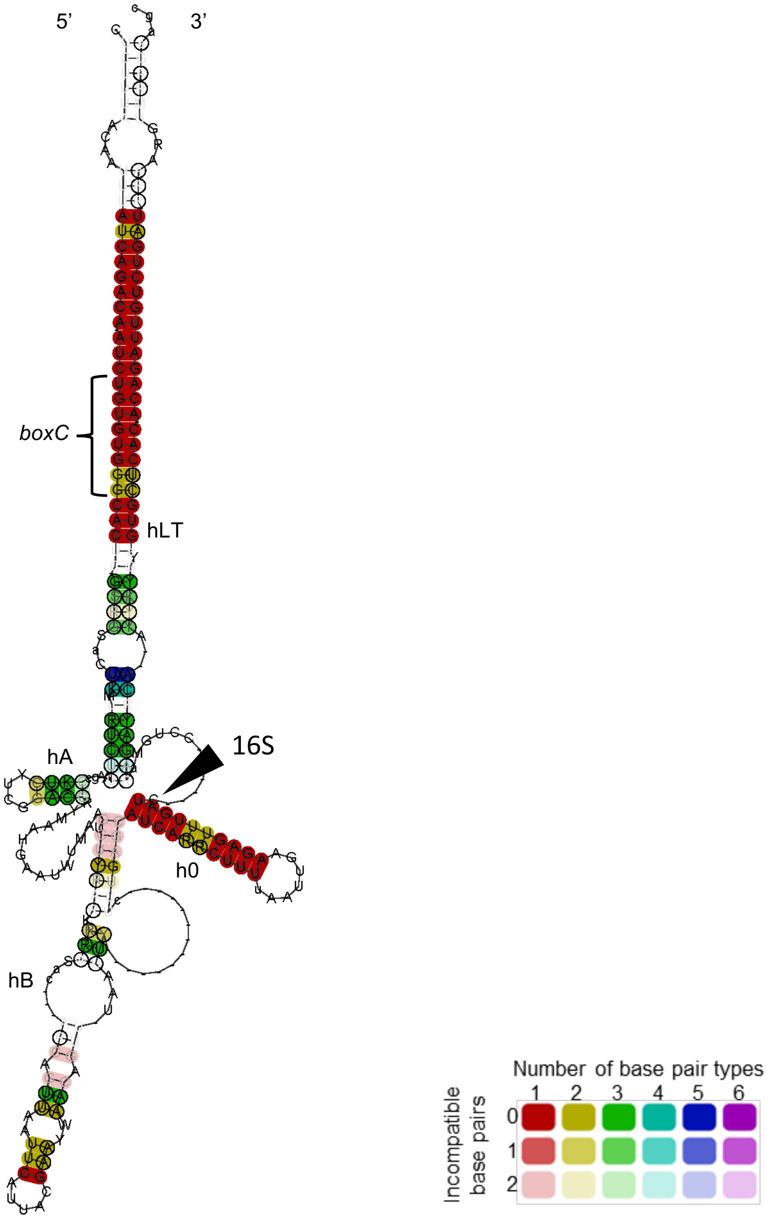

Figure S7. Secondary structure derived for the *Cronobacter* clade (node: N825, n=138) of Enterobacteriaceae.

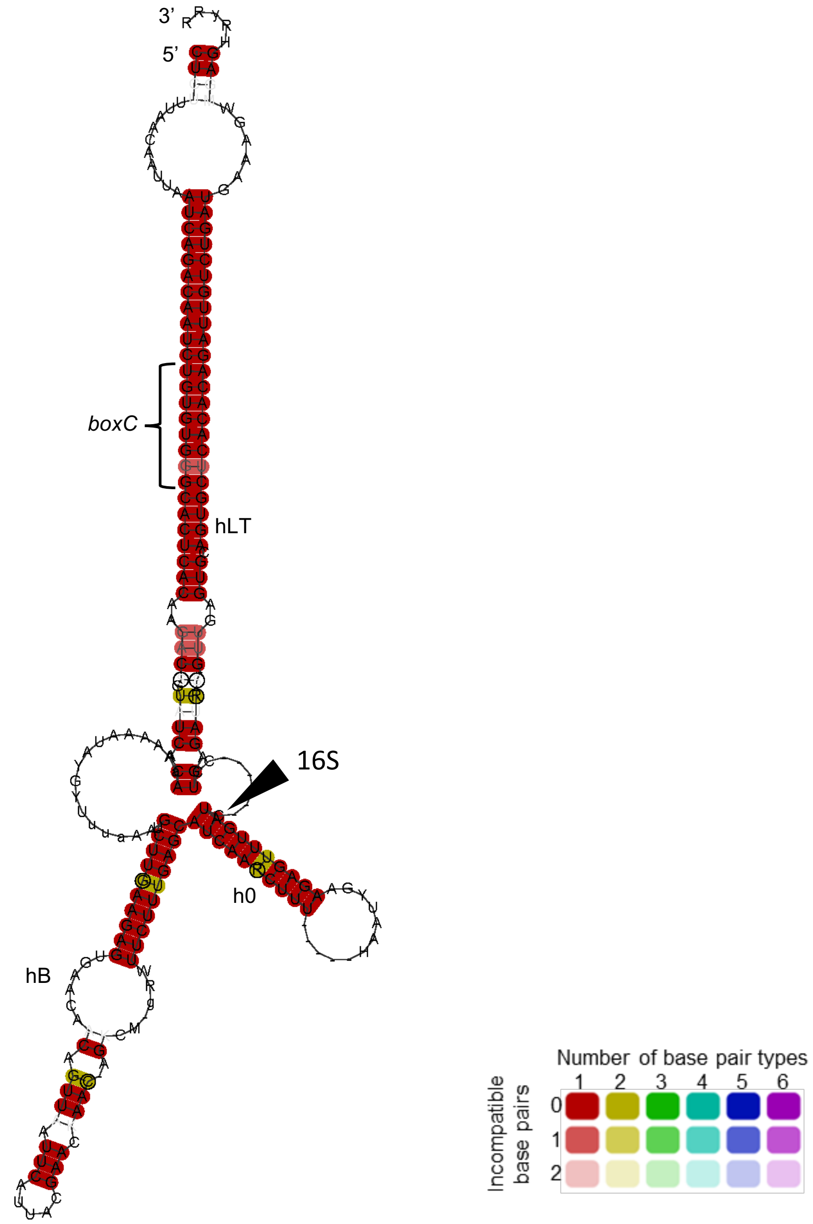

Figure S8. Secondary structure derived for the *Photorhabdus* clade (node: N855, n=30) of Enterobacteriaceae.

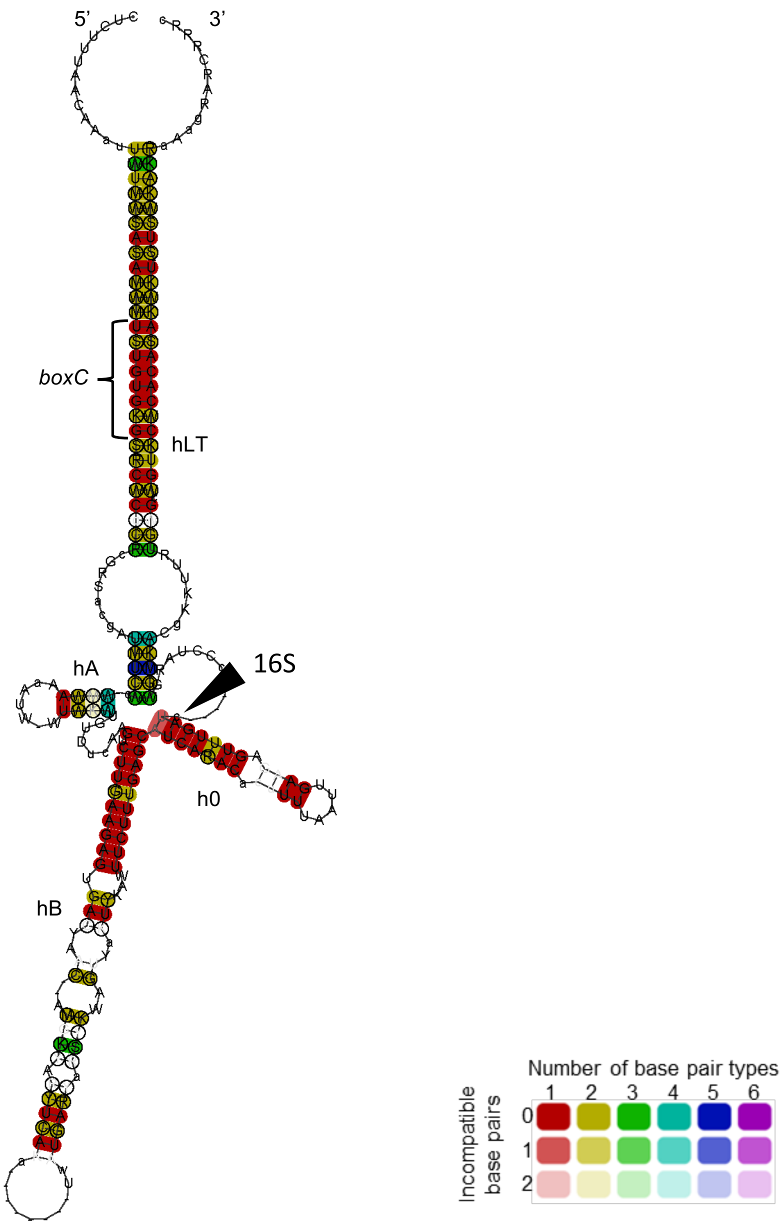

Figure S9. Secondary structure derived for the *Proteus* clade (node: N907, n=51) of Enterobacteriaceae.

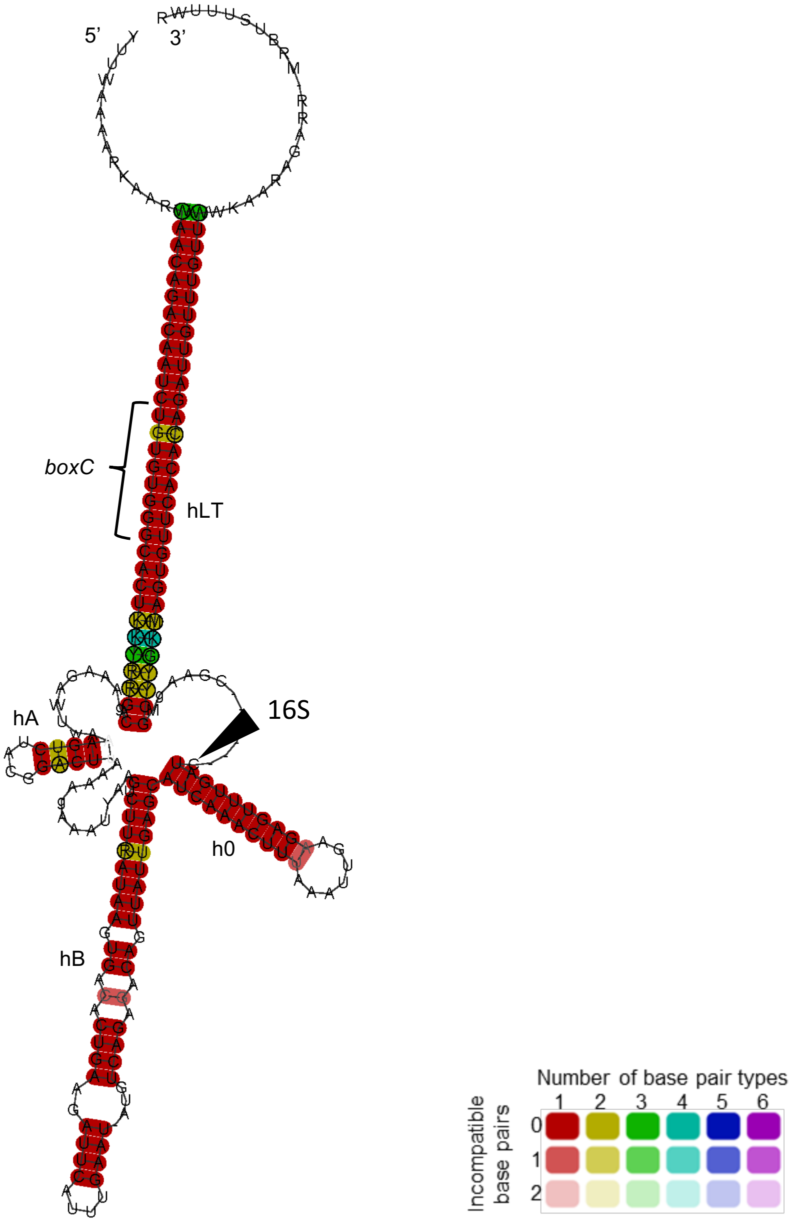

Figure S10. Secondary structure derived for the *Frischella* clade (node: N916, n=9) of Enterobacteriaceae.

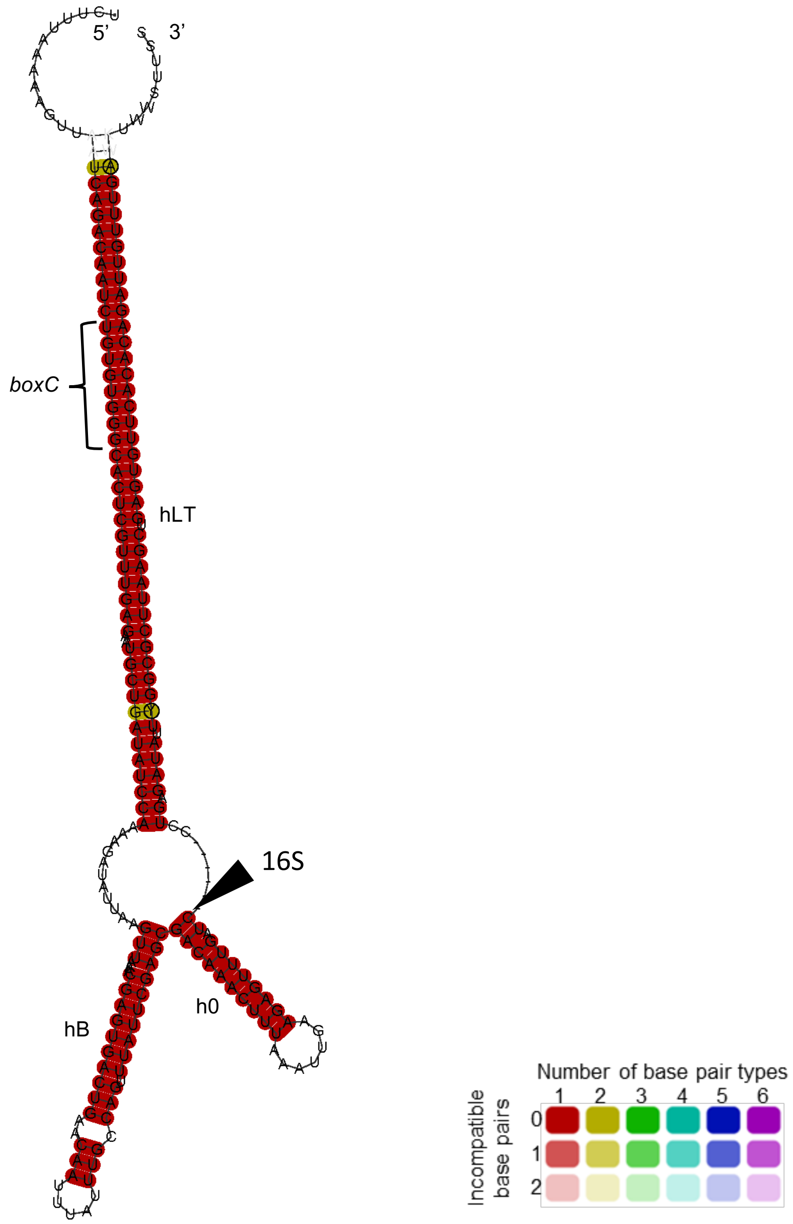

Figure S11. Secondary structure derived for the *Plesiomonas* clade (node: N927, n=11) of Enterobacteriaceae.

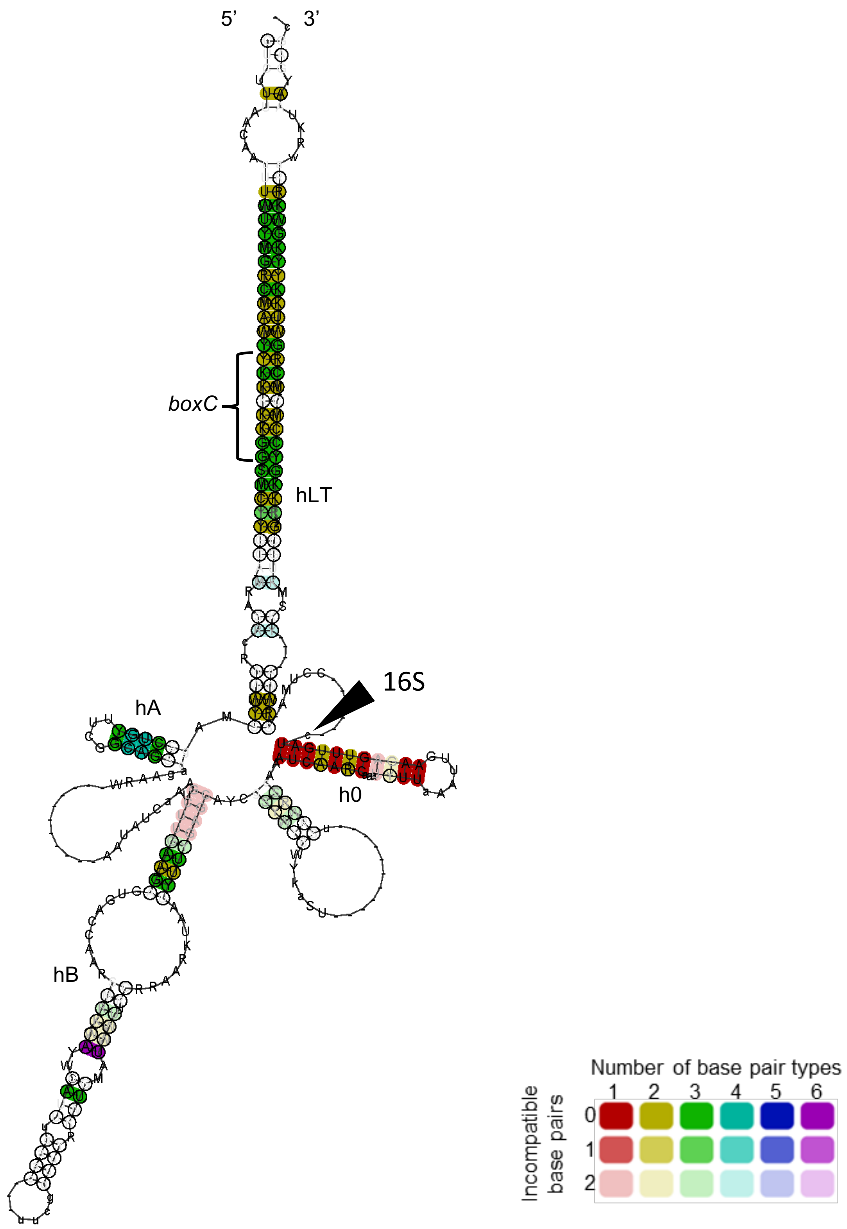

Figure S12. Secondary structure derived for the *Yersinia* clade (node: N1144, n=217) of Enterobacteriaceae.

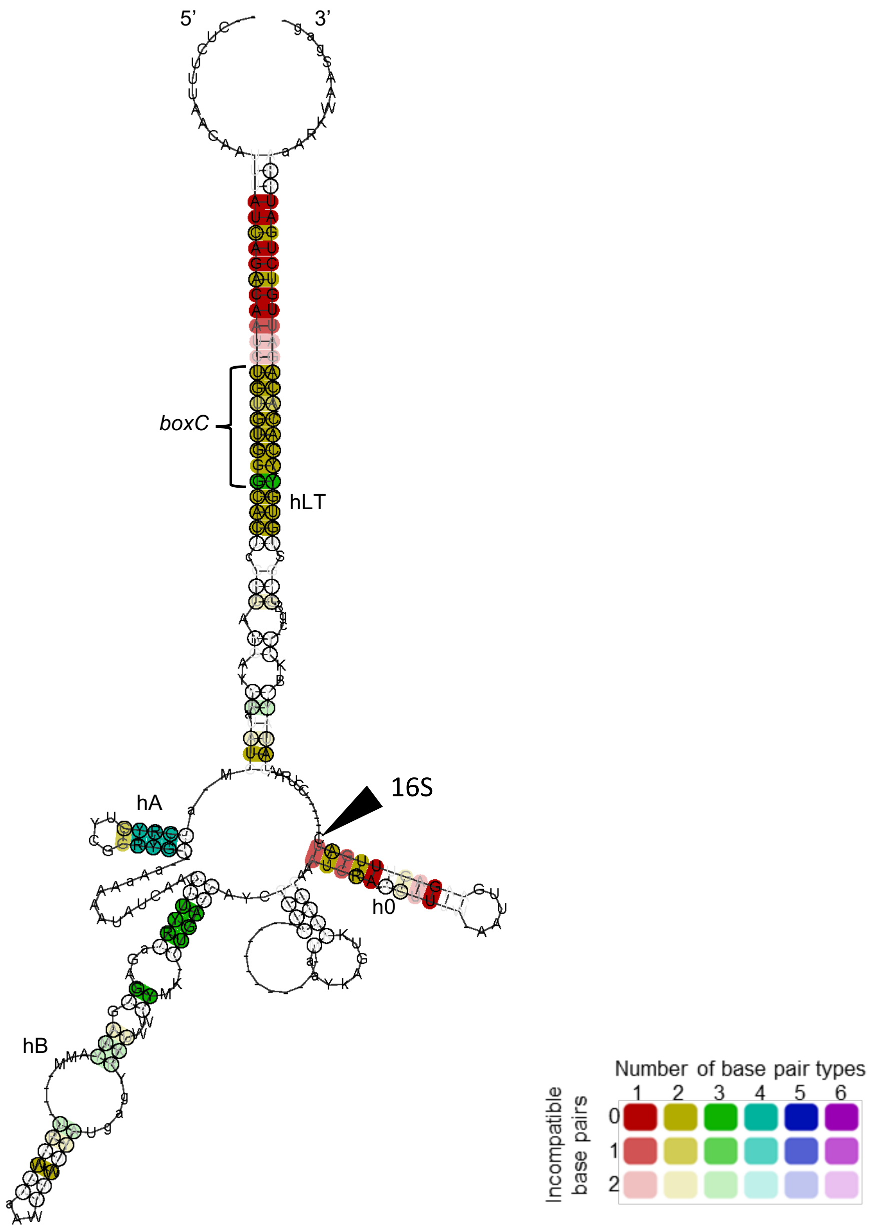

Figure S13. Secondary structure derived for the *Serratia* clade (node: N1278, n=135) of Enterobacteriaceae.

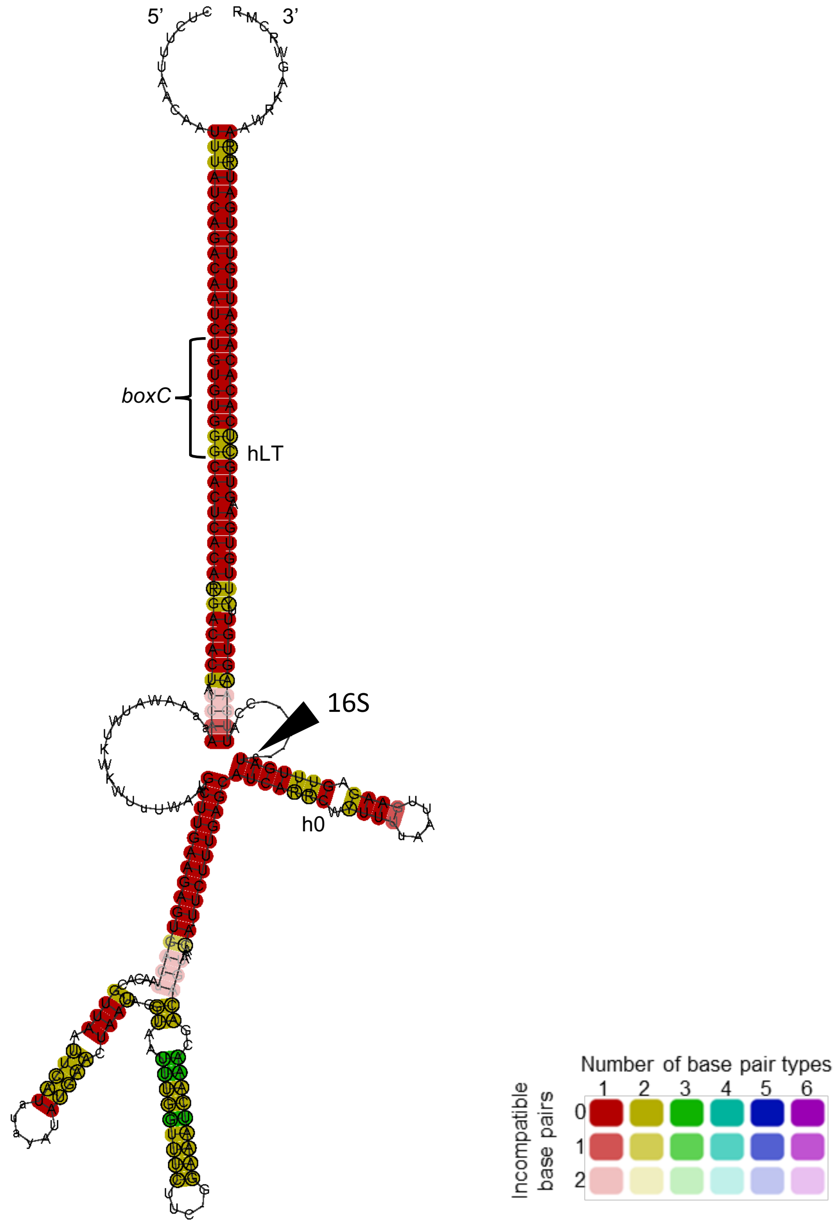

Figure S14. Secondary structure derived for the *Providencia* I clade (node: N1311, n=31) of Enterobacteriaceae.

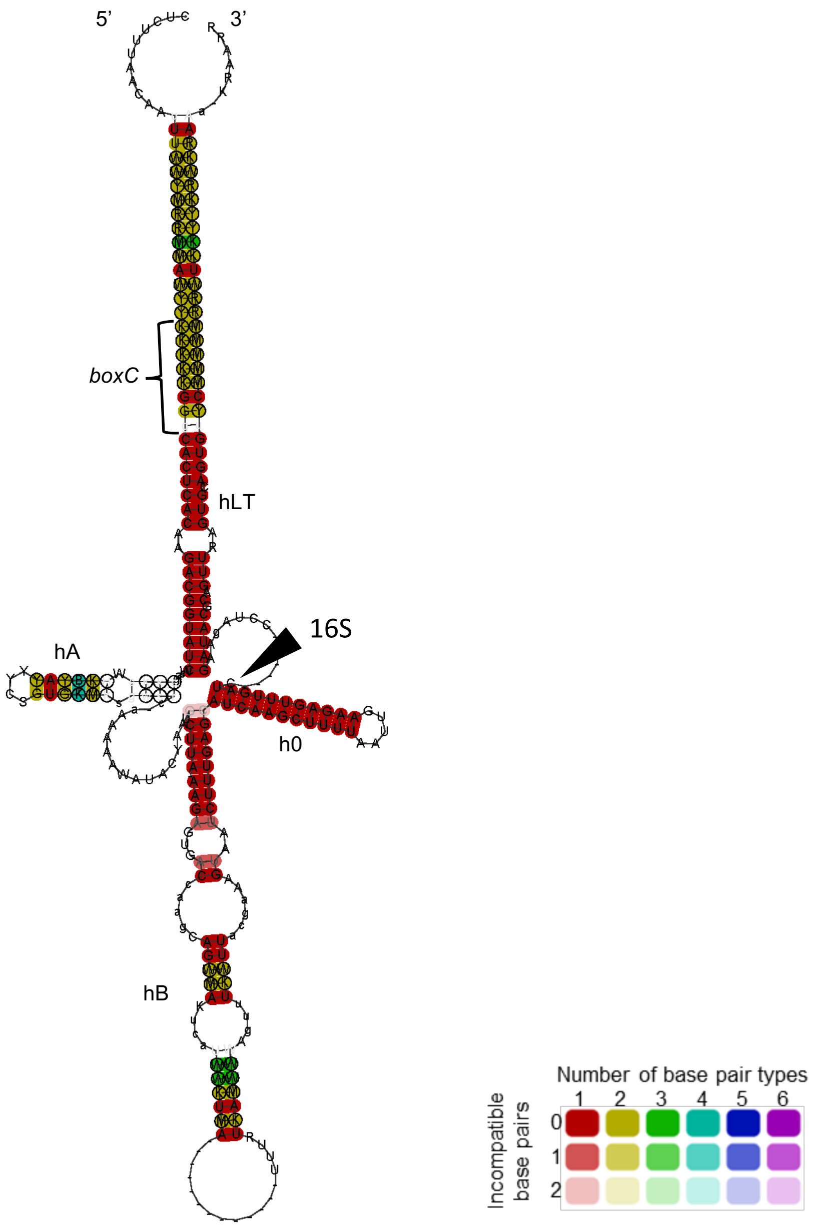

Figure S15. Secondary structure derived for the *Budvicia* clade (node: N1325, n=15) of Enterobacteriaceae.

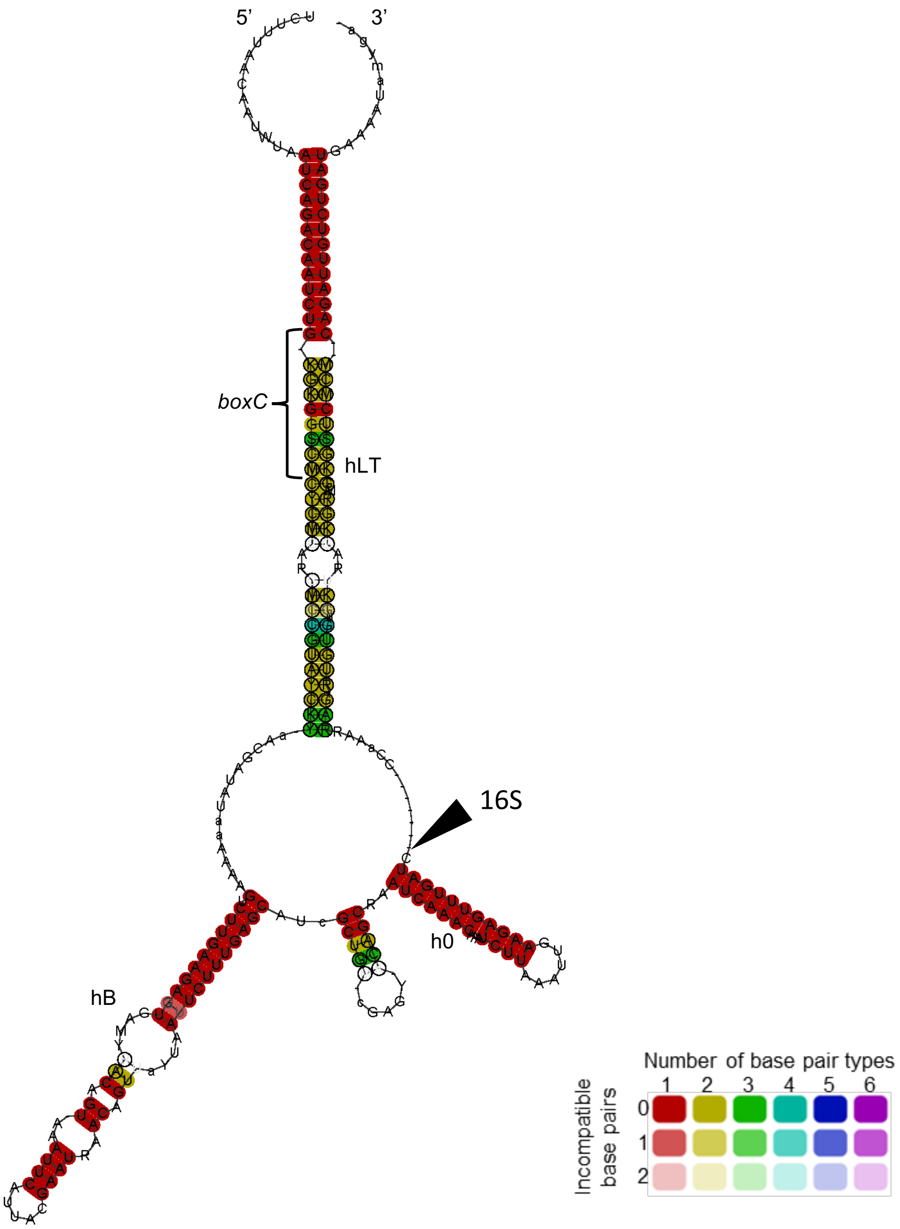

Figure S16. Secondary structure derived for the *Pectobacterium* clade (node: N1394, n=68) of Enterobacteriaceae.

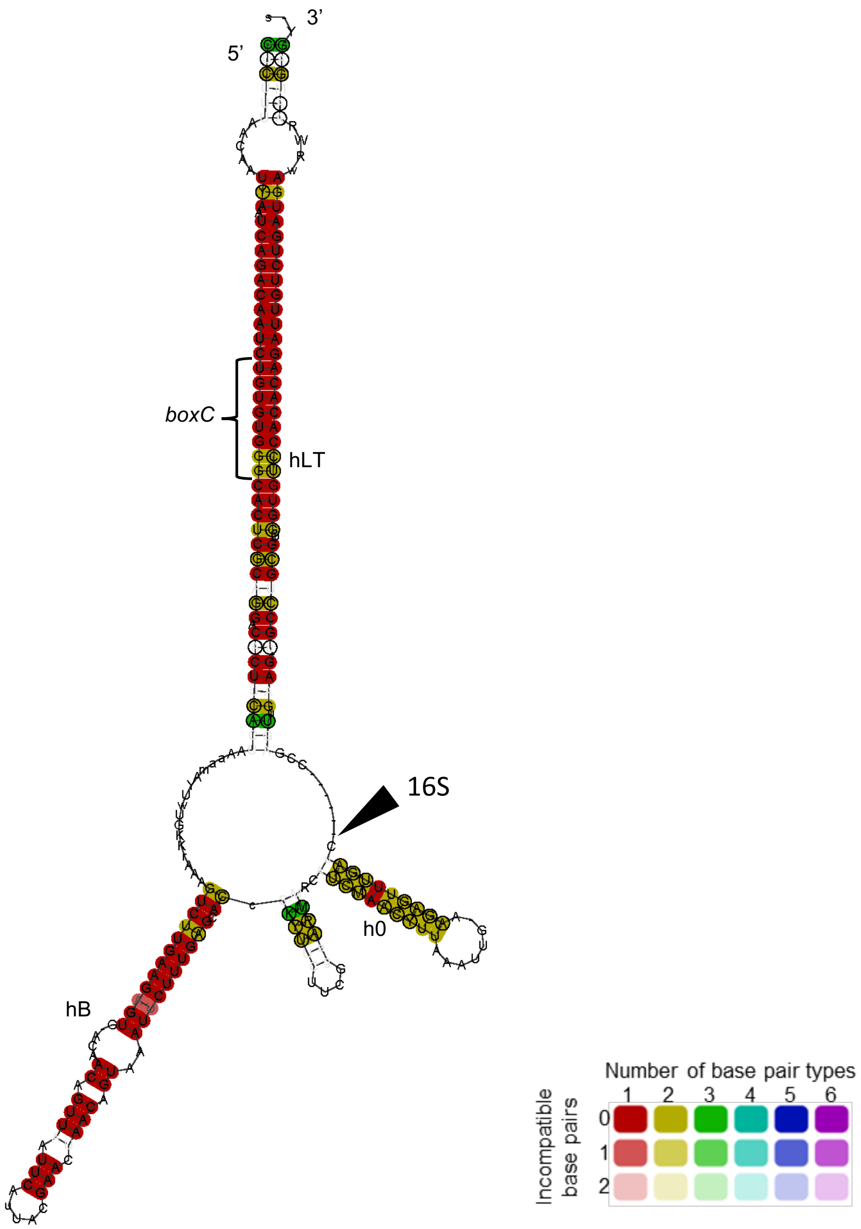

Figure S17. Secondary structure derived for the *Dickeya* clade (node: N1432, n=39) of Enterobacteriaceae.

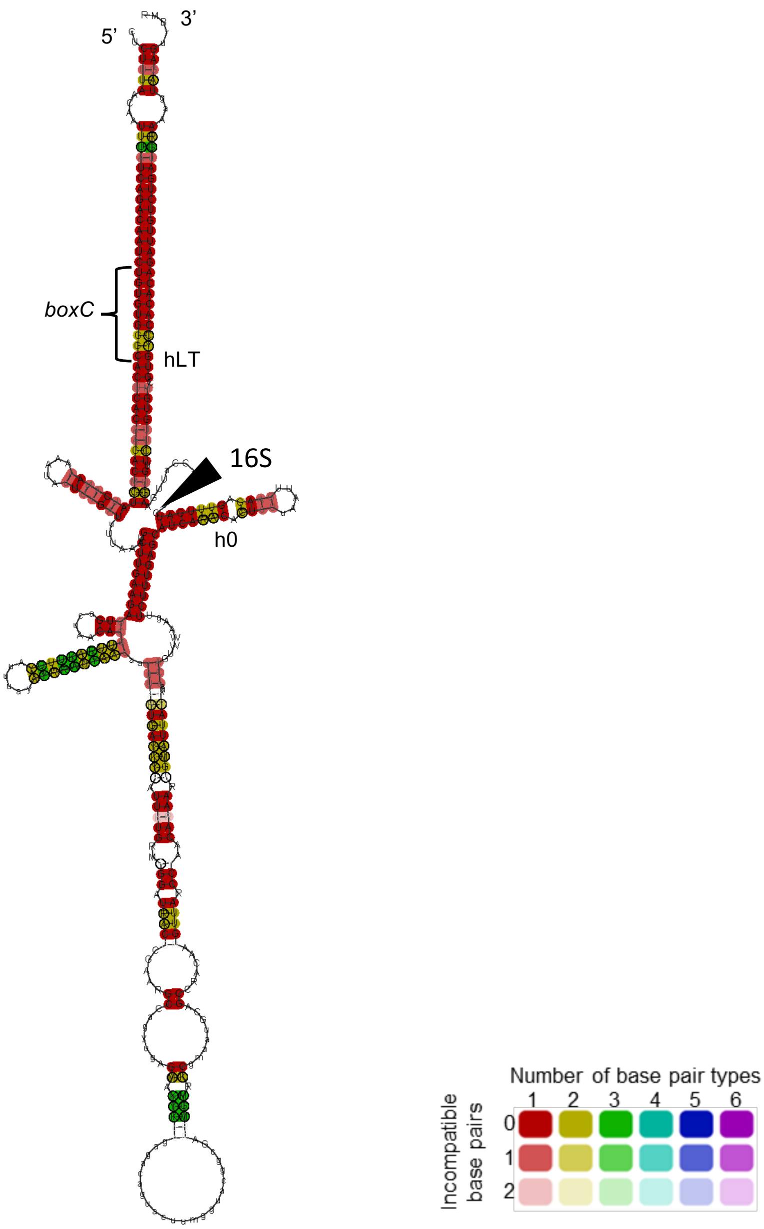

Figure S18. Secondary structure derived for the *Providencia* II clade (node: N1441, n=6) of Enterobacteriaceae.

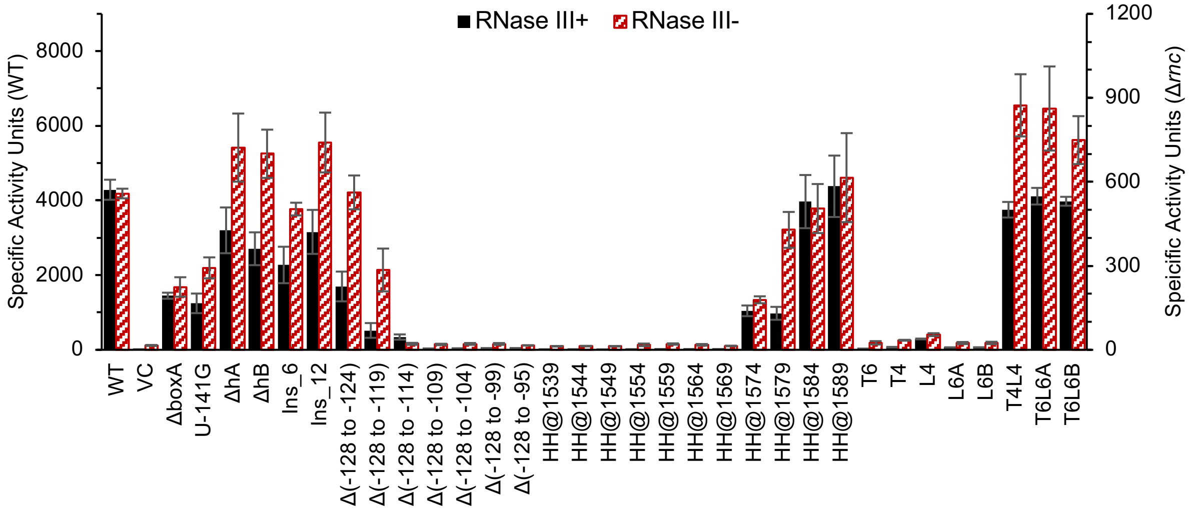

Figure S19. Effects of mutations on translation activity are independent of RNase III. Translation activities of ribosomes made from various mutant constructs (as indicated) in *rnc*+ and Δ*rnc* cells. Black filled bars represent activity (left axis) of constructs in the *rnc*+ indicator strain (KLF2674). Red hash-filled bars represent activity (right axis) of constructs in the Δ*rnc* indicator strain (BRW299). Data represent the mean ± SEM from three or more biological replicates.

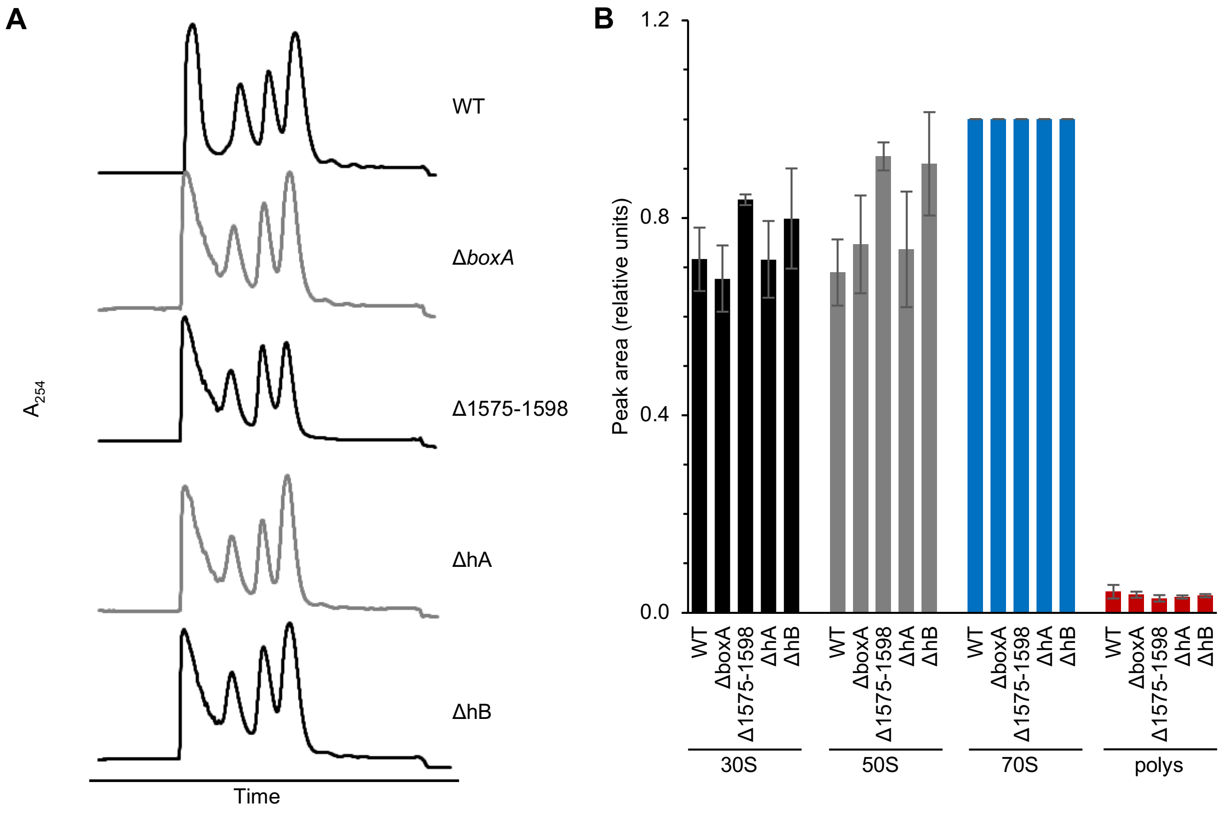

Figure S20. Sucrose gradient sedimentation analysis of Δ7 prrn strains harboring various leader / trailer mutations. (A) Representative A_{254} traces of sucrose gradients of Δ7 prrn (Rec^{-}) strains (as indicated). (B) Quantification of the 30S (black), 50S (grey), and polysome (red) peaks, relative to the 70S (blue) peak. Data represent the mean ± SEM of three biological replicates.

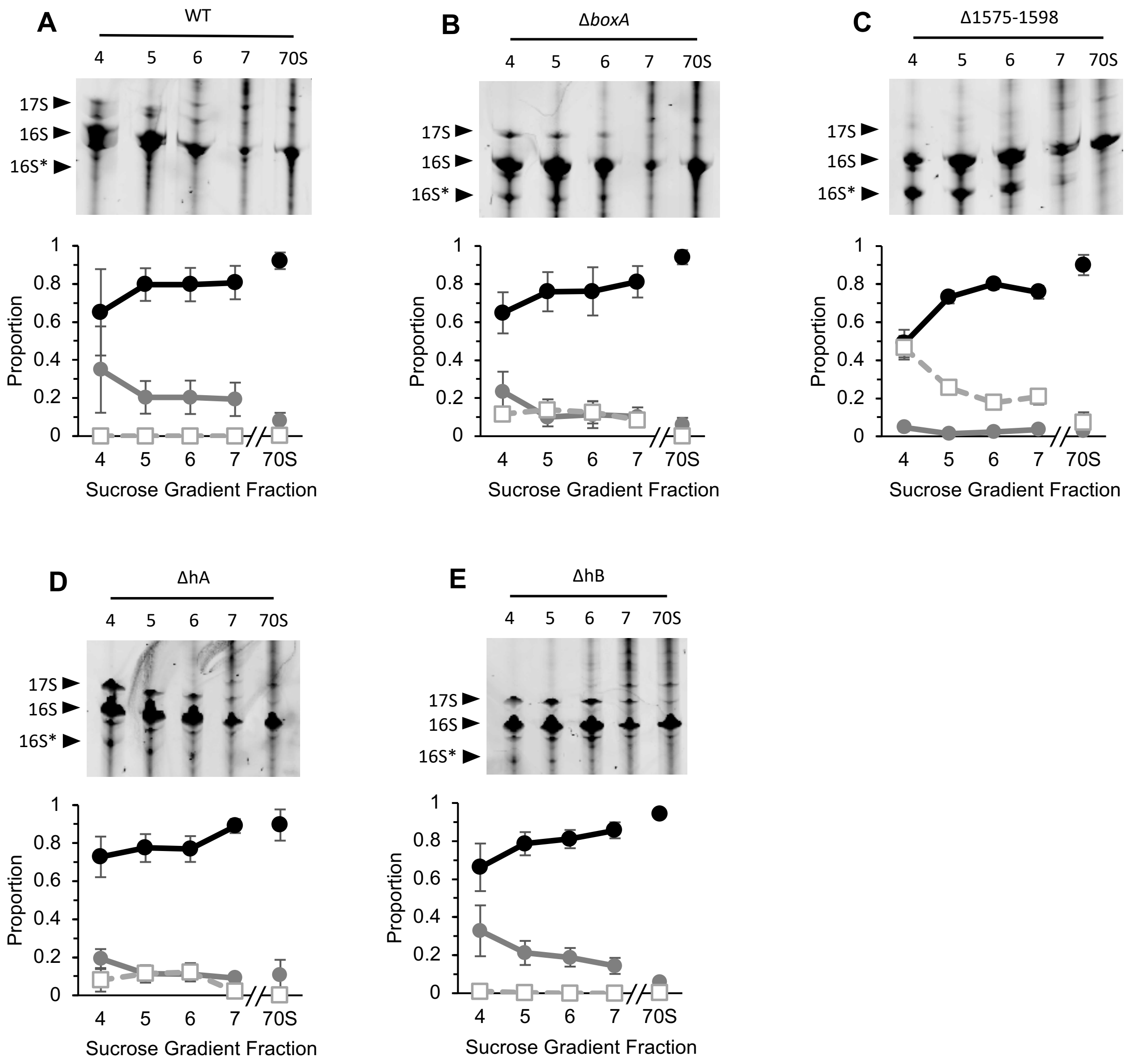

Figure S21. Small subunit particles contain a shortened RNA species in several mutant Δ7 prrn strains. (A-E top panel) RNA was extracted from various sucrose gradient fractions and analyzed by PAGE. A representative gel (top) and quantified data (bottom) are shown for WT (A), Δ*boxA* (B), Δ1575-1598 (C), ΔhA (D), and ΔhB (E) strains. Bands corresponding to 17S, 16S, and 16S* are indicated. Fractions 4-5 correspond to the pre-30S region and 6-7 to the 30S region. Fractions 11-12 (containing the 70S ribosome peak) were pooled together. In plots, data points represent the mean ± SEM of three biological replicates. Black filled circles, 16S rRNA; grey filled circles, 17S rRNA; white open squares, 16S* rRNA.

Table S2. Plasmids used in this study

Plasmid	Reference	Description
p278MS2	(1)	pBR322 derivative containing the *rrnB* operon under control of the λ P_L promoter.
pDQ207	(2)	pBAD18 containing *rrsB*, encoding 16S rRNA with ASD 5'-GGGAU-3'
pBW022	This work	pDQ207 containing marker mutation T1451A
pBW023	This work	pBW022 encoding pre-16S rRNA with deletion of nt -209 to -1 (ΔL)
pBW024	This work	pBW022 encoding pre-16S rRNA with nt 1543-1545 replaced by a hammerhead ribozyme (ΔT), predicted to cleave between nt 1544 and 1545.
pBW025	This work	pBW024 encoding pre-16S rRNA with deletion of nt -209 to -1 (ΔLΔT)
pBW039	This work	pBW022 with an SpeI-XhoI fragment encoding the hammerhead inserted at *rrsB* position 1612.
pBW044	This work	pBW039 encoding pre-16S rRNA with deletion of nt 1540 to 1618 (HH@1539)
pBW045	This work	pBW039 encoding pre-16S rRNA with deletion of nt 1545 to 1618 (HH@1544)
pBW046	This work	pBW039 encoding pre-16S rRNA with deletion of nt 1550 to 1618 (HH@1549)
pBW047	This work	pBW039 encoding pre-16S rRNA with deletion of nt 1555 to 1618 (HH@1554)
pBW048	This work	pBW039 encoding pre-16S rRNA with deletion of nt 1560 to 1618 (HH@1559)
pBW049	This work	pBW039 encoding pre-16S rRNA with deletion of nt 1565 to 1618 (HH@1564)
pBW050	This work	pBW039 encoding pre-16S rRNA with deletion of nt 1570 to 1618 (HH@1569)
pBW051	This work	pBW039 encoding pre-16S rRNA with deletion of nt 1575 to 1618 (HH@1574)
pBW052	This work	pBW039 encoding pre-16S rRNA with deletion of nt 1580 to 1618 (HH@1579)
pBW053	This work	pBW039 encoding pre-16S rRNA with deletion of nt 1585 to 1618 (HH@1584)
pBW054	This work	pBW039 encoding pre-16S rRNA with deletion of nt 1590 to 1618 (HH@1589)
pBW061	This work	pBW022 encoding pre-16S rRNA with deletion of nt -128 to -1 [ΔL(+*boxBA*)]
pBW062	This work	pBW022 encoding pre-16S rRNA with deletion of nt -209 to -133 (Δ*boxBA*)
pBW063	This work	pBW022 encoding pre-16S rRNA with deletion of nt -86 to -75 (ΔhA)
pBW065	This work	pBW022 encoding pre-16S rRNA with deletion of nt -59 to -12 (ΔhB)
pBW075	This work	pBW039 encoding pre-16S rRNA with 5'-AAAUUU-3' inserted in between nt -133 and -134 (Ins_6)
pBW076	This work	pBW039 encoding pre-16S rRNA with 5'-UCUAGAAAAUUU-3' inserted in between nt -133 and -134 (Ins_12)
pBW079	This work	pBW039 encoding pre-16S rRNA with deletion of nt -128 to -124 (Δ-128 to -124)
pBW080	This work	pBW039 encoding pre-16S rRNA with deletion of nt -128 to -119 (Δ-128 to -119)
pBW081	This work	pBW039 encoding pre-16S rRNA with deletion of nt -128 to -114 (Δ-128 to -114)
pBW082	This work	pBW039 encoding pre-16S rRNA with deletion of nt -128 to -109 (Δ-128 to -109)
pBW083	This work	pBW039 encoding pre-16S rRNA with deletion of nt -128 to -104 (Δ-128 to -104)
pBW084	This work	pBW039 encoding pre-16S rRNA with deletion of nt -128 to -99 (Δ-128 to -99)
pBW085	This work	pBW039 encoding pre-16S rRNA with deletion of nt -128 to -95 (Δ-128 to -95)
pBW086	This work	pBW039 encoding pre-16S rRNA with deletion of nt -209 to -145 (Δ*boxB*)
pBW087	This work	pBW039 encoding pre-16S rRNA with deletion of nt -147 to -133 (Δ*boxA*)
pBW088	This work	p278MS2 encoding pre-16S rRNA with deletion of nt -209 to -145 (Δ*boxB*)
pBW089	This work	p278MS2 encoding pre-16S rRNA with deletion of nt -147 to -133 (Δ*boxA*)
pBW090	This work	p278MS2 encoding pre-16S rRNA with deletion of nt -209 to -133 (Δ*boxBA*)
pBW091	This work	pBW039 encoding pre-16S rRNA with deletion of nt -209 to -129 (Δ-209 to -129)
pBW092	This work	pBW039 encoding pre-16S rRNA with deletion of nt -209 to -124 (Δ-209 to -124)
pBW093	This work	pBW039 encoding pre-16S rRNA with deletion of nt -209 to -119 (Δ-209 to -119)
pBW094	This work	pBW039 encoding pre-16S rRNA with deletion of nt -209 to -114 (Δ-209 to -114)
pBW095	This work	pBW039 encoding pre-16S rRNA with deletion of nt -209 to -108 (Δ-209 to -108)
pBW122	This work	pBW039 encoding pre-16S rRNA with nt 1568 to 1575 changed to 5'-GGCAGUUC-3' (T6)
pBW123	This work	pBW039 encoding pre-16S rRNA with nt 1568 to 1575 changed to 5'-CUCAGUUC-3' (T4)
pBW124	This work	pBW039 encoding pre-16S rRNA with nt -117 to -110 changed to 5'-GAACUGUC-3' (L6A)
pBW125	This work	pBW039 encoding pre-16S rRNA with nt -117 to -110 changed to 5'-GAACUGGG-3' (L4)
pBW126	This work	pBW039 encoding pre-16S rRNA with nt -117 to -110 changed to 5'-GAACUGCC-3' (L6B)
pBW127	This work	pBW122 encoding pre-16S rRNA with nt -117 to -110 changed to 5'-GAACUGUC-3' (T6L6A)
pBW128	This work	pBW123 encoding pre-16S rRNA with nt -117 to -110 changed to 5'-GAACUGGG-3' (T4L4)
pBW129	This work	pBW124 encoding pre-16S rRNA with nt 1568 to 1575 changed to 5'-GGCAGUUC-3' (T6L6A)
pBW130	This work	pBW125 encoding pre-16S rRNA with nt 1568 to 1575 changed to 5'-CUCAGUUC-3' (T4L4)
pBW131	This work	pBW126 encoding pre-16S rRNA with nt 1568 to 1575 changed to 5'-GGCAGUUC-3' (T6L6B)
pBW132	This work	pBW122 encoding pre-16S rRNA with nt -117 to -110 changed to 5'-GAACUGCC-3' (T6L6B)
pBW133	This work	p278MS2 encoding pre-16S rRNA with deletion of nt 1543 to 1598 (Δ1543 to 1598)
pBW134	This work	p278MS2 encoding pre-16S rRNA with deletion of nt 1570 to 1598 (Δ1570 to 1598)
pBW135	This work	p278MS2 encoding pre-16S rRNA with deletion of nt 1571 to 1598 (Δ1571 to 1598)
pBW136	This work	p278MS2 encoding pre-16S rRNA with deletion of nt 1575 to 1598 (Δ1575 to 1598)
pBW137	This work	p278MS2 encoding pre-16S rRNA with deletion of nt -86 to -75 (ΔhA)
pBW138	This work	p278MS2 encoding pre-16S rRNA with deletion of nt -61 to -12 (ΔhB)
pBW141	This work	pBW039 encoding pre-16S rRNA with mutation U-141G

(1) Youngman E., Brunelle J., Kochaniak A., Green R. (2004) *Cell*, **117**, 589-599
(2) Qin D. and Fredrick K. (2009) *Molecular Microbiology*, **71**, 1239-1249

Table S3. Conservation of *boxC* in family Enterobacteriaceae.

boxC sequence	Number of sequences
UCUGUGUGGG	1431
UCUGUGUGGA	3
UCUGUGUGGC	3
UUUGUGUGGG	2
UCUGCGUGGA	1
AAUGUGUGGG	1

Table S4. Spacing between *boxA* and *boxC* in the Enterobacteriaceae.

Distance[a]	Number of sequences
16	1
17	2
18	1287
19	121
20	6
21	4

[a]Number of nucleotides between *boxA* and *boxC*.
