# Supplementary material for: Roles of the leader-trailer helix and antitermination complex in biogenesis of the 30S ribosomal subunit
Source: Nucleic Acids Res. 2023 Apr 27;51(10):5242–54. doi: 10.1093/nar/gkad316 (PMC10250234; doi:10.1093/nar/gkad316)
Supplement: gkad316_Supplemental_Files [file Fig_S1.pdf › Fig_S1.pdf]

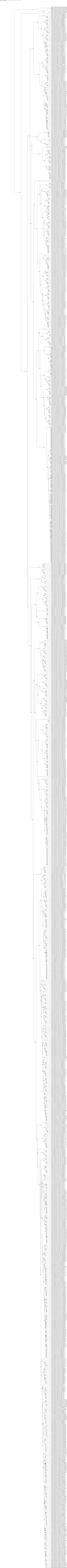
Tree scale: 10000
